# Strategies to promote uptake and use of intimate partner violence and child maltreatment knowledge: an integrative review

**DOI:** 10.1186/1471-2458-14-862

**Published:** 2014-08-21

**Authors:** Jennifer CD MacGregor, Nadine Wathen, Anita Kothari, Prabhpreet K Hundal, Anthony Naimi

**Affiliations:** Faculty of Information & Media Studies, The University of Western Ontario, North Campus Building, Room 240, 1151 Richmond St., London, ON N6A 5B7 Canada; Faculty of Health Sciences, The University of Western Ontario, Arthur and Sonia Labatt Health Sciences Building, Room 222, 1151 Richmond St., London, ON N6A 5B9 Canada; Schulich Interfaculty Program in Public Health, The University of Western Ontario, The Western Centre for Public Health and Family Medicine, 1465 Richmond St., 4th Floor, London, ON N6G 2M1 Canada; Lab for Knowledge Translation in Health, The University of Western Ontario, Arthur and Sonia Labatt Health Sciences Building, Room 403, 1151 Richmond St., London, ON N6A 5B9 Canada

**Keywords:** Intimate partner violence, Child maltreatment, Knowledge translation, Integrative review, Evidence-based

## Abstract

**Background:**

Intimate partner violence (IPV) and child maltreatment (CM) are major social and public health problems. Knowledge translation (KT) of best available research evidence has been suggested as a strategy to improve the care of those exposed to violence, however research on how best to promote the uptake and use of IPV and CM evidence for policy and practice is limited. Our research asked: 1) What is the extent of IPV/CM-specific KT research? 2) What KT strategies effectively translate IPV/CM knowledge? and 3) What are the barriers and facilitators relevant to translating IPV/CM-specific knowledge?

**Methods:**

We conducted an integrative review to summarize and synthesize the available evidence regarding IPV/CM-specific KT research. We employed multiple search methods, including database searches of Embase, CINAHL, ERIC, PsycInfo, Sociological Abstracts, and Medline (through April, 2013). Eligibility and quality assessments for each article were conducted by at least two team members. Included articles were analyzed quantitatively using descriptive statistics and qualitatively using descriptive content analysis.

**Results:**

Of 1230 identified articles, 62 were included in the review, including 5 review articles. KT strategies were generally successful at improving various knowledge/attitude and behavioural/behavioural intention outcomes, but the heterogeneity among KT strategies, recipients, study designs and measured outcomes made it difficult to draw specific conclusions. Four key themes were identified: existing measurement tools and promising/effective KT strategies are underused, KT efforts are rarely linked to health-related outcomes for those exposed to violence, there is a lack of evidence regarding the long-term effectiveness of KT interventions, and authors’ inferences about barriers, facilitators, and effective/ineffective KT strategies are often not supported by data. The emotional and sometimes contested nature of the knowledge appears to be an important barrier unique to IPV/CM KT.

**Conclusions:**

To direct future KT in this area, we present a guiding framework that highlights the need for implementers to use/adapt promising KT strategies that carefully consider contextual factors, including the fact that content in IPV/CM may be more difficult to engage with than other health topics. The framework also provides guidance regarding use of measurement tools and designs to more effectively evaluate and report on KT efforts.

**Electronic supplementary material:**

The online version of this article (doi:10.1186/1471-2458-14-862) contains supplementary material, which is available to authorized users.

## Background

Intimate partner violence (IPV) and child maltreatment (CM) are recognized as major social and public health problems [1, 2], with serious physical and mental health consequences for individuals [3–7], as well as devastating consequences for families, communities, and even the economy e.g., [8–11]. Data about the scope and impact of IPV and CM began to coalesce in the latter part of the 20^th^ century, coinciding with the increased focus on evidence-based practice and policy in health and social services [12, 13]. It was not surprising, therefore, that the new millennium brought attention to the relative lack of evidence-based interventions available to decision-makers in IPV and CM across the spectrum from clinical care, social service provision, and policy development [14, 15]. This led to increasing calls to conduct rigorous research to better understand how IPV and CM can be effectively identified, addressed, and, ideally, prevented [16–18]. The last decade has seen a marked increase in the number and types of research studies addressing both IPV and CM e.g., [7, 10, 17, 19, 20], and many systematic evidence reviews (of various types) synthesizing what is known (and not known) regarding specific interventions for women, children, and men, and from the perspective of victims and perpetrators e.g., [6, 17, 21, 22]. As highlighted in recent reviews of the evidence for effective interventions in CM [23–26], and IPV [6, 15, 20, 27–29], the two fields differ in terms of the amount and nature of research evidence available to inform practice and policy [30], with considerably more interventions having been tested and shown effective or promising in the CM literature, as compared to the IPV literature. A growing challenge is how best to promote the uptake and use of the best available evidence in each of the CM and IPV fields, as well as emerging evidence at their intersection, such as interventions for children exposed to IPV [31–33], or those that take a lifespan approach, recognizing that earlier violence exposures can have consequences at later life stages [18, 30]. In parallel, the past decade has seen exponential growth in research on the issue of ‘knowledge translation’ (KT) [34, 35] or ‘implementation science’ [36]. However, research specific to understanding how best to promote the uptake and use of IPV and CM evidence for policy, advocacy and practice is more limited [37]. This integrative review summarizes and synthesizes the available evidence regarding IPV- and CM-specific KT research, and provides suggestions about the design of future KT initiatives.

### Knowledge translation

In the 1990s, the advent of the evidence-based medicine (EBM) movement in the UK, Canada and the USA [12, 38] brought the idea of KT into mainstream clinical practice, where it quickly spread to other areas of health and social service delivery and policy [39–41]. Early KT research focused on identifying the prevalent and persistent gaps between research and practice – health innovations are slow to make their way into common practice and, as a result, patients often do not receive the best possible care [42–45]. Attention was then focused on developing and testing KT strategies to address these gaps, with significant descriptive work across different practice and policy contexts e.g., [46, 47]. Despite the proliferation of KT research, KT continues to undergo an ‘identity crisis’ of sorts [40]. Part of the problem is in terminology [48, 49]. Although we have chosen to use the term KT, many other terms are used in the literature to describe the same or similar processes (or parts of the process), such as: implementation science, knowledge mobilization, research utilization, knowledge transfer, knowledge exchange, and dissemination, just to name a few. Moreover, no single agreed upon definition exists and the conceptualization of KT has evolved over time. Whereas it was once considered primarily about addressing the research to *practice* gap (i.e., focused on how to get the best available evidence into the hands of health care providers), recently it has been defined more broadly to address the *knowledge* to *action* gap, thus expanding the range of potential knowledge users to health policymakers, health advocates, or the general public, for example [39, 50]. Another change to the conceptualization of KT expands the definition of knowledge. For many, the ‘K’ in ‘KT’ is synonymous with ‘research evidence’. However, some have questioned whether research evidence is indeed the only relevant knowledge worth translating to potential users [51, 52]. For example, local knowledge and tacit knowledge are highly valued by many health and social service providers [53, 54].

A third way in which KT has been redefined over time relates to the process itself. Rather than involving one-way, linear processes whereby knowledge creators deliver neatly packaged ‘knowledge’ to potential users, as is the case with traditional ‘science push’ (i.e., researcher driven) and ‘demand pull’ (i.e., user driven) models, more nuanced approaches are increasingly favoured [55]. Particularly in the case of complex topics and knowledge use contexts (e.g., policy development) [56, 57], the concept of knowledge translation *and exchange* (an ‘interaction’ model) is more relevant, denoting that knowledge users and their needs significantly shape the interaction and must be addressed to ensure more effective knowledge uptake and use [55, 58–60].

Finally, a fourth way in which KT has matured is through a focus on understanding the ways that best practices/guidelines and/or specific proven-effective interventions can become part of routine clinical practice or sustained within an organizational setting [61, 62]. This process, generally called implementation science, is also concerned with scaling up innovative local practices for broader, lasting practice change. Implementation science draws from the original Diffusion of Innovations theory to identify early adopters and champions of new practices as a way to promote scaling up at a systems level [63]. One topic of interest is finding the right balance between fidelity and adaptation, since attempts to effect change using a ‘one-size-fits-all’ strategy are generally unsuccessful and highlight the need to take relevant contextual, cultural, and policy factors into account [64]. Implementation science is also concerned with the need to re-evaluate or develop strong criteria for external validity – traditional research provides knowledge about what works under controlled conditions but often lacks information about how to make interventions work within complex social systems [65]. Interested readers are directed to Fixen et al.’s detailed review of the literature in the area [66].

For the purposes of this review, KT is defined as the process of strategically moving (by various methods) knowledge (broadly defined) from those who create, synthesize, or have access to it, to those who can use it, for the purpose of improving decision-making. Embedded in this definition are several key points related to the evolution of the conceptualization of KT as described above. First, knowledge users are those who can use knowledge, with ‘use’ in this instance defined broadly to include any of the following three established forms: instrumental (i.e., direct, concrete use), symbolic (i.e., to confirm decisions already made or justify thinking) or conceptual (i.e., indirect or diffuse use, e.g., to assist in understanding) [67]. The contexts of use are also broadly defined, and include service delivery, policy, planning and/or advocacy efforts. Second, ‘knowledge’ is not restricted to research evidence, but also includes, for example, tacit and contextual knowledge [53]. Third, KT need not, but certainly can, involve a process of exchange with knowledge users.

### Rationale and research questions

As better evidence regarding how to effectively identify, treat, and prevent IPV and CM (apart and collectively) emerges [16, 17, 24], researchers need guidance regarding how to best translate this knowledge. At the same time, knowledge users can benefit from an understanding of “research-pull” strategies and mechanisms that could be useful as they are increasingly expected to bring research evidence into their decision-making processes. Similarly, when research evidence exists but is contentious, as in the example of the effectiveness of universal IPV screening [17, 68], KT can be particularly challenging [37, 69], and knowledge of how best to communicate “difficult” evidence under these conditions, and in complex areas such as IPV and CM, is necessary. This integrative review updates, summarizes and synthesizes IPV- and CM-specific KT literature.

The review, due to its broad definition of knowledge and knowledge users, includes KT strategies applicable to a wide range of knowledge use contexts, from community-based settings to clinical settings [53]. Topic-specific KT reviews are indeed becoming more popular e.g., [70, 71], perhaps because of recognition that ‘one size does not fit all’ when it comes to KT [37, 53, 59]. We believe that family violence is a unique context requiring focused attention to appropriate KT practices that demonstrate value, while minimizing potential harms.

To our knowledge, one published review on KT in the area of IPV and CM exists. Larrivée, Hamelin-Brabant, and Lessard [72] reviewed 22 papers, including 13 empirical research and 9 non-empirical papers (e.g., commentaries). Their review is a valuable contribution to the literature, in particular the authors’ attention to both theoretical and applied work in this area. Our review builds upon this work by conceptualizing KT more broadly, thus allowing for the inclusion of a wider range of relevant research to inform IPV and CM decision-making. This approach is in keeping with Mitton et al. [46], authors of a highly-cited KT review, who acknowledged that work labeled differently can still make valuable contributions to the KT literature.

The present research is designed to answer three key questions: 1) What is the extent of IPV- and CM-specific KT research? 2) What KT strategies effectively translate IPV and/or CM knowledge? and 3) What are the barriers and facilitators relevant to translating IPV- and CM-specific knowledge? Integrative review methodology [73, 74] was used because it systematically summarizes and synthesizes literature to generate new knowledge in an area of study and can reveal gaps in the literature to highlight future research priorities [75]. This type of review is also amenable to diverse forms of research evidence (quantitative and qualitative studies) and can be used for large or small bodies of literature [74].

## Method

### Search strategy

Four search methods were used [74, 76]. First, with the guidance of expert research librarians, we conducted searches using the Medline, Embase, CINAHL, PsycINFO, Sociological Abstracts, and ERIC databases. In each database, we used a combination of subject headings (i.e., controlled vocabulary) and/or keywords in the title or abstract. We harvested key terms by examining papers that discussed KT or IPV/CM terminology [39, 48, 77], by examining the subject headings and key words used in the databases, and relying on experts in the field to ensure we included important terms. Over 30 KT keywords were included in each search, including: knowledge translation, knowledge mobilization, research utilization, guideline implementation, information uptake, and dissemination (for a summary of all database search terms, see Additional file 1). Over 20 IPV/CM keywords were used, including: partner abuse, wife battering, child neglect, family violence and spouse abuse. Original database searches were completed in August 2012; no date restrictions were imposed. An updated search was conducted at the end of April 2013. Second, a research assistant conducted hand-searches of some key IPV and CM journals (e.g., *Journal of Interpersonal Violence, Child Abuse & Neglect*) going back three years (2009-2011). Third, we examined the reference lists of articles included in the review. Finally, we contacted several researchers in the area of IPV and/or CM to inquire as to whether or not they knew of any additional articles that should be included in our review.

### Inclusion criteria

Only articles published in English and in their entirety (as opposed to abstracts only) were evaluated for inclusion in the review. We did not limit our review to any particular type of study design – quantitative, qualitative and mixed method research were eligible for inclusion. Unpublished works (e.g., conference abstracts, dissertations, etc.) were excluded.

Two team members independently examined the title and abstract of retrieved articles to determine whether they met five inclusion criteria (see Table 1). First, the article had to describe research involving KT, as defined above. Therefore, the KT had to be planned or strategic, as opposed to ‘naturally occurring’, such as an analysis of IPV research covered in the media or knowledge exchange taking place in journal clubs e.g., [78, 79]. It could involve the translation of knowledge that did not necessarily (but could) take the form of research evidence, and could or could not involve a process of mutual knowledge exchange with potential knowledge users. Second, the research had to evaluate outcomes related to knowledge uptake and/or utilization, or conduct a process evaluation of KT implementation efforts. Thus, research examining either conceptual/symbolic use (i.e., changes in self-reported knowledge, awareness, attitudes, or abilities) and/or instrumental use (i.e., changes in behaviour or behavioural intention) met this inclusion criterion.

**Table 1:** **Criteria for study inclusion**

Criteria	Included	Excluded
**Sample/KT ‘recipients’**	• Health care professionals (e.g., physicians, nurses, etc.)	• General public
		• IPV/CM victims and perpetrators
		• Trainees (e.g., medical students, undergraduates)
	• NGOs/community organizations (e.g., IPV/CM-related service providers)	
	• Decision/policymakers	
	• Other (e.g., teachers, law enforcement)	
**Focus/Intervention**	• KT is focused on IPV and/or CM	• Therapy training
	• KT must be planned/strategic	• Parenting skills training
**Outcomes**	• Knowledge/attitudes (including awareness, beliefs, self-efficacy, etc.)	None
	• Behavioural outcomes (intention to use or actual use of knowledge)	
	• Clinical outcomes	
	• Process outcomes	
**Study design**	All	None
**Language**	English	All other languages
**Publication type**	Peer-reviewed, scholarly literature	Grey literature, sources unavailable in full-text
(Table adapted from Stacey et al., [71])		

Third, the KT had to focus on IPV- and/or CM-related knowledge. Finally, the ‘recipients’ of the KT were required to be health care or social service providers, educators, NGO/community-based organization staff, including advocates, or decision/policy-makers. Initiatives designed to translate knowledge to other recipients, for example general awareness campaigns targeted at the public, were not included e.g., [80]. We also did not include research focused on translating knowledge to potential victims of abuse so that they might protect themselves (i.e., safety planning), or to abusers so that they might discontinue their abusive behaviour, as these topics are the focus of other reviews [14, 21, 22, 81, 82]. Evaluations of the effectiveness of parenting skills training programs, particular types of abuse-related therapies or therapy training programs (e.g., trauma-focused cognitive behavioural therapy) have also been addressed elsewhere and are outside the scope of this review [83–85]. Research focused on translating knowledge to trainees (e.g., medical students) was excluded, however continuing professional education was included, since it is directed at practicing health or social service providers.

The two reviewers compared and discussed their inclusion decisions. When in doubt, the article was put forward to the full-text review phase.

### Quality appraisal

As with inclusion decisions, each article was independently assessed for quality by two members of the research team. If a decision could not be reached, the article was discussed with a senior team member for input. To evaluate the quality of existing literature reviews relevant to our topic, we used the AMSTAR tool, which has been demonstrated to be reliable and valid [86]. Because our review was not limited to experimental designs, and included a variety of types of systematic reviews (e.g., realist-informed) [87], AMSTAR criteria specific to statistical meta-analyses were not used (i.e., were given a rating of ‘not applicable’). Ultimately, each article received a ranking of poor, fair, or good, with the latter two moving to the data extraction phase.

Our quality appraisal of primary articles was guided by a tool designed to simultaneously appraise the quality of quantitative, qualitative, and mixed method research [88]. For ease of use, we adapted the tool by combining its items into key categories (rationale, sampling and recruitment, intervention and outcomes, data collection and analyses). The extent to which an article met Sirriyeh et al.’s criteria was assessed and a qualitative ranking of poor, fair, or good was assigned.

### Data extraction, coding and analysis

Information (e.g., study design, findings, etc.) was extracted from each included article independently by one research team member and entered into separate data extraction forms. Key data points were checked for accuracy against the original articles by the first author.

To address our research question regarding the extent of IPV/CM KT research, we used SPSS 20.0 to run descriptive statistics (e.g., determine the number of articles with a particular research design, etc.). To address our research questions designed to synthesize findings regarding effective KT strategies and barriers/facilitators, we used a qualitative, thematic approach. All data extraction forms for included articles were imported into NVivo 10.0 software. Following Whittemore and Knafl’s [74], p. 550 recommendation to “simplify, abstract, focus, and organize data into a manageable framework”, the first author coded key study findings and made ongoing notes for each article and research question. Key codes used included: study findings (by type of outcomes measured, knowledge/attitudes, behavioural, etc.), intervention approach (e.g., passive presentation, complex/multifaceted, etc.), effective/ineffective intervention components, and barriers/facilitators. This process allowed the extracted data to be viewed in an organized way and, using an iterative process, examined to “identify patterns, themes, or relationships” [74], p. 551. Key patterns were verified by the first author and discussed with the second author, an expert in the area of IPV.

## Results

### Inclusion and quality appraisal

**Figure 1:**
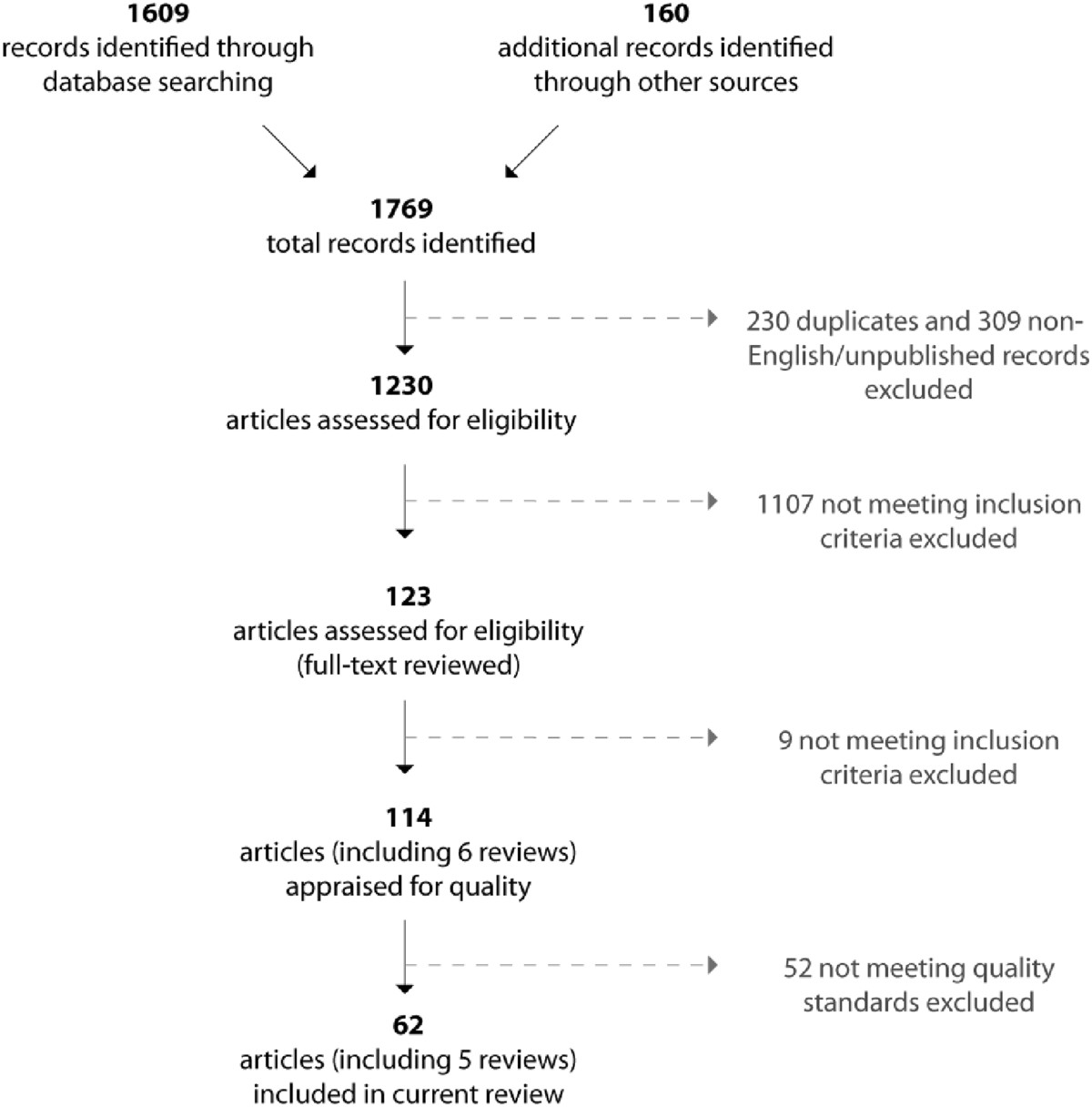
**Search Flow Diagram.**

In total, 1230 articles were considered for inclusion and 123 articles appearing to meet all five criteria (including 6 systematic reviews) were independently reviewed by two team members; 11 of these were resolved by consensus decision of the full research team. The final pool of articles for quality appraisal was 114, including 6 systematic reviews. For a summary of the results of our searches, please see Figure 1 (adapted from PRISMA) [76].

In keeping with recommendations in the literature [89], we calculated both percent agreement and a kappa statistic to assess inter-rater reliability of the quality appraisals. Overall, substantial agreement was achieved, as indicated by 83% agreement and a kappa statistic of .75 [89, 90]. For 10 articles, another team member was consulted to reach a quality decision.

All but one of 6 systematic reviews met a minimum standard of fair quality. Our primary articles were compared against these reviews and, to avoid findings being double-counted, if already contained in a review, were excluded from our analysis. The review deemed to be poor was not included in our review, but primary articles within it were assessed for inclusion. Among the 108 primary articles appraised, 57 met the minimum standard of fair quality and were included in our review (see Additional file 2 for a complete reference list of articles included in this review, Additional file 3 for a detailed summary of included articles, and Additional file 4 for a summary of articles not meeting quality standards). The main weaknesses of articles assigned a ‘poor’ rating were: small sample sizes/low response rates, inadequate descriptions of measurement tools, use of poor measurement tools, inadequate/unclear reporting of statistical analyses, and insufficient details regarding the study method or intervention.

### The extent of IPV and CM-specific KT research

#### General Characteristics

Articles originating in the US were most common, and most were published in 2006 or later (see Table 2 for article characteristics). Of the 57 primary articles, the vast majority used a quantitative approach (n = 47, 82%), some used mixed methods (n = 9, 16%), and only one was purely qualitative (2%). Many research designs were used (see Table 2). The majority of the articles included in the reviews were also quantitative in nature. Recipients of the KT strategies were most often health practitioners but half involved other types of recipients, often, but not always in non-healthcare settings (see Table 2).

**Table 2:** Characteristics of included Articles (N = 62*)

Article Characteristic	n (%)*
**Country of origin**	
US	37 (60)
Canada	8 (13)
UK	7 (11)
Australia	3 (5)
Other (e.g., the Netherlands)	7 (11)
**Publication year**	
2006 or later	32 (52)
Between 2000 and 2005	18 (29)
Before 2000	12 (19)
**Study design**	
Experiment/RCT	17 (30)*
Pre-post	19 (33)*
Quasi-experiment (i.e., comparative analysis with no randomization to groups)	9 (16)*
Other (e.g., post-test only)	12 (21)*
**Focus**	
IPV	22 (36)
CM	28 (45)
IPV and CM	12 (19)
**KT ‘Recipients’**	
Health practitioners (e.g., physicians, dentists etc.)	31 (50)
Teachers/educators	5 (8)
‘Social’ practitioners (e.g., child protection workers)	3 (5)
Other (e.g., law guardians/attorneys)	2 (3)
Combination of Above**	21 (34)

*Percentages are out of 62 (the total number of included articles) unless indicated with an asterisk (in which case the denominator is 57, the total number of included primary articles). ** Over half of which included health practitioners.

#### Types of interventions

Only a few of the articles used KT terms as the primary way to describe the intervention (n = 2, 3%). Most used the term ‘training’ in some way (e.g., ‘in-service training’; n = 33, 53%), while others used education-related terms (e.g., ‘educational intervention’; n = 13, 21%), ‘program’ (e.g., ‘support program’; n = 5, 8%) or something else specific to the intervention (e.g., ‘interactive multi-media tutorial’) or ‘intervention’ more generally (n = 9, 15%).

In a review on domestic violence training programs published over a decade ago [91], Davidson et al. found that training often involved single sessions of 1-3 hours. We found the interventions to be much more varied, and often more thorough. Training sessions (or workshops, computer-based courses, etc.) ranged in length from 30 minutes to over a week in total, were often accompanied (or replaced entirely) by other components, and varied greatly with regard to the breadth, depth and mode of knowledge translation. Therefore, to synthesize the range of different types of interventions, we categorized the interventions in primary articles into six broad types (see Table 3). The least common type was training involving only passive presentation of knowledge (5% of articles; e.g., lecture). One of the most common types was multi-mode without knowledge exchange (26%). These interventions were characterized by single or multiple training sessions involving multiple ‘modes’ of delivery (e.g., video, role-play, etc.), but did not have an ‘exchange’ component whereby recipients interacted with facilitators (e.g., researchers, other experts) to engage with the knowledge. Multimode with exchange was also fairly common (23%).

**Table 3:** **Effectiveness of interventions, by type, in the 62 reviewed articles**

Intervention type n(%) studies addressing	Definition	Articles (first author, year)	Overall effectiveness of intervention type
Primary Articles			
**Passive presentation** 3(5%)	Passive/didactic presentation of knowledge (e.g., in training session or workshop)	Aved, 2007; Cross, 2007; Lia-Hoagberg, 1999	Generally effective at improving knowledge/attitude and behaviour/behavioural intention outcomes (few studies and varied outcomes, however, warrant cautious interpretation)
**Multimode without exchange** 16(28%)	Single or multiple training sessions involving multiple ‘modes’ of delivery (e.g., video, role-play, etc.), but no exchange between recipients and facilitators	Botash, 2005; Darby, 2007; Harris, 2011; Harris, 2002; Hibbard, 1987; Hsieh, 2006; Jones, 2004; Knapp, 2006; McGrath, 1987; Paranal, 2012; Protheroe, 2004; Short, 2006; Smeekens, 2011; Sullivan, 1990; Walker, 2009; Young, 2008	Generally effective at improving knowledge/attitude and behavioural outcomes, evidence for knowledge/attitude outcomes is stronger
**Multimode with exchange** 12(21%)	Single or multiple training sessions involving multiple ‘modes’ of delivery (e.g., video, role-play, etc.), including exchange between recipients and facilitators	Allert, 1997; Barber-Madden, 1983; Davila, 2006; Hazzard, 1984; Kleemeier, 1988; Lo Fo Wong, 2006; McCosker, 1999; Nicolaidis, 2005; Salmon, 2006; Schoening, 2004; Shefet, 2007; Wathen, 2011	Generally effective at improving knowledge/attitude and behavioural outcomes, evidence for knowledge/attitude outcomes is stronger
**Complex/multifaceted** 15(26%)	Intensive, multi-component interventions, usually over extended period of time	Berger, 2002; Bonds, 2006; Campbell, 2001; Cerezo, 2004; Cyr, 2009; Dresser, 2012; Dubowitz, 2011; Feder, 2011; Heyman, 2009; Janssen, 2002; Paluzzi, 2000; Rischke, 2011; Thompson, 2000; Whitaker, 2012; Zachary, 2002	Generally effective at improving knowledge/attitude and behaviour/behavioural intention outcomes (but most behavioural evidence is for screening or identification rates)
**Other** 6(11%)	Interventions do not fit clearly into above categories or multiple intervention types are compared	Boursnell, 2010; Chaffin, 1994; Lamb, 2000; Olson, 1996; Rheingold, 2012; Socolar, 1998	*
**Unclear/unknown** 5(9%)	In most cases, training duration, but not format, is known	Agirtan, 2009; Hawkins, 2001; Khan, 2005; Saunders, 2005; Warburton, 2006	*
**Review articles**			
**Varied** 5(100%)	All review articles included studies using varied intervention types	Davidson, 2001; Larrivée, 2012; Louwers, 2010; Newton, 2010; O’Campo, 2011	*

*Relevant findings from articles categorized as ‘Other’, ‘Unclear/unknown’ and ‘Varied’ were incorporated into syntheses for the other four intervention types above. Further details for all included studies are provided in Additional file 3.

Complex/multifaceted interventions were also common (26%). These interventions were intensive, involved multiple components, and usually occurred over an extended period of time. They often involved training sessions in conjunction with organization-level components such as: a champion or dedicated staff member to provide ongoing support, chart prompts to encourage IPV screening, intervention tailoring based on staff feedback or observation, and changes to the environment (e.g., posters). Complex/multifaceted interventions often, but not always, included an exchange component. Examples include ongoing telephone support from the researchers e.g., [92] and in-person performance feedback from a consultant e.g., [93].

Other types of interventions included ‘other’ (i.e., did not fit clearly into any other category; 11%), and unclear/unknown (i.e., insufficient detail prevented categorization^a^; 9%). There did not seem to be any association between the focus of the paper (IPV, CM or both) and the type of intervention.

#### Outcomes

Of the 62 articles, the majority reported on both knowledge/attitude and behavioural/behavioural intention outcome variables (n = 36, 58%); others reported on behavioural/behavioural intention outcomes only (n = 17, 27%) or on knowledge/attitude outcomes only (n = 9, 15%). Measurement and conceptualization of knowledge/attitude-related constructs varied greatly. Some authors measured self-reported change, current level of, or confidence in, knowledge, whereas others compared group or pre-post differences in performance on tests of knowledge. Included in this category are also related constructs such as ‘self-efficacy’ (e.g., perceptions of abilities to carry out a particular behaviour), ‘beliefs’, ‘attitudes’, and ‘perceptions’ (e.g., beliefs about knowledge level, attitudes toward particular IPV or CM-related issues, etc.), and ‘awareness’ (usually indistinguishable from ‘knowledge’). Knowledge/attitude outcomes ranged in their level of specificity, with some focused on particular types of knowledge relevant in specific contexts (e.g., awareness of site-specific CM policies and procedures) or for specific roles (e.g., attitudes toward IPV screening in the ED), whereas others measured knowledge more broadly (e.g., prevalence and importance of IPV, IPV myths, signs of CM, etc.), or measured multiple types of IPV and/or CM-related knowledge.

Conceptualizations and measurement of behavioural/behavioural intention outcomes were also varied. The most common outcomes related to the ‘detection’ or ‘identification’ of CM or IPV victims. For example, many reported IPV inquiry and/or identification rates from chart audits, rates of self-reported IPV enquiry, or the quality of IPV or CM case documentation. Examples of other behavioural outcomes include: performance on actual or simulated interviews with suspected CM victims, actual or self-reported filed reports of suspected CM, and self-reported development of IPV or CM-related policies and procedures. Interestingly, some behavioural outcomes actually involved further KT, such as the number of IPV training sessions held or the number of multidisciplinary teams formed for child protection (i.e., teams that went on to hold conferences etc.) [94].

A relatively small number of articles (n = 16) devoted any attention to evaluation of some aspect of the intervention process, but thorough process evaluation was rare. Examples of process variables include: participant perceptions of adequacy of emotional support during web training, attendance at training, and anecdotal accounts of how well training was received by participants.

Virtually no articles reported on health outcomes – that is, outcomes evaluating how the health or wellbeing of suspected, potential, or actual victims of violence or maltreatment was impacted by the KT. Proxy outcomes included screening and/or disclosure rates e.g., [87], patient satisfaction with clinical care [95], women’s perceptions of caseworker helpfulness [96], and actual service usage (e.g., substance abuse service use in child protection cases) [97].

### Effective KT strategies

In general, interventions designed to improve IPV/CM-related knowledge/attitude outcomes were successful; that is, it was rare for authors to report that the intervention had no effect on the knowledge/attitude outcomes that were measured. Across the range of conceptualizations and approaches to measurement of knowledge/attitude outcomes, a variety of different intervention types were found to be effective (for a summary of effectiveness by intervention type, see Table 3; see Additional file 3 for a detailed summary of all findings from included articles). However, because the types of knowledge/attitudes were so varied, as were the methods used to measure them, it is impossible to distinguish which strategies were most effective to mobilize certain kinds of knowledge, and to what effect. It appears that a variety of interventions, ranging in complexity, time commitment, etc., can be successful. Only one article [98] warned that brief training – providing practitioners with a little knowledge regarding how to respond to IPV victims – could actually cause false confidence, and ultimately be potentially harmful to victims. However, this possibility was not empirically examined in any article.

Interventions also tended to be successful in improving the behavioural and behavioural intention outcomes that they were designed to improve, although findings from the review papers tended to be more mixed. Complex/multifaceted interventions were always used in studies designed to assess behaviour change (either on its own or in addition to knowledge/attitude measures), suggesting some fit between the intensity of an intervention and the goals it sought to achieve. In their review of 17 IPV screening programs, O’Campo and colleagues [87] concluded that ‘comprehensive’ strategies (i.e., ones involving multiple program components and institutional support) were more likely to be successful than non-comprehensive strategies in increasing screening and identification rates. Interestingly, however, complexity isn’t always necessary; the simple addition of a chart prompt (a component of a KT intervention that we would not consider ‘KT’ itself) appears to be effective at increasing IPV and CM-related inquiry e.g., [99, 100].

Generally speaking, however, it is difficult to draw conclusions regarding specific components of interventions that are effective. We found that the majority of articles did not use a study design capable of distinguishing effective components of interventions or modes of delivery (e.g., video vs. role-play). When authors did identify successful components or modes, their conclusions often appeared to be based on unsystematic observation, informal participant feedback, or general findings drawn from the literature, rather than on specific and valid data.

With regard to their successful implementation of a complex program to increase agreement on diagnostic decisions regarding child abuse, Heyman et al. [101], p. 909 stated “[d]ismantling studies would need to be conducted to isolate which elements are necessary to achieve high levels of agreement.” Such acknowledgement regarding the lack of understanding of crucial intervention components was rare in our sample. Nonetheless, several components, repeatedly endorsed in our sample (albeit with varying degrees of evidence to support them), show *promise*: ongoing initiatives such as repeated training e.g., [87] or continued monitoring of KT efforts and desired outcomes e.g., [102], opportunities for exchange with experts or personal reflection and discussion between recipients e.g., [37, 103] and components that make the organizational culture more conducive to KT e.g., [104, 105].

Despite the success of many different KT strategies to improve IPV/CM knowledge/attitudes and behaviour/behavioural intention, the majority of studies evaluate improvements only in the relative short-term, a finding consistent with Davidson et al.’s [91] conclusion over a decade ago. Overall, we found that 6-month and 1 to 3-year follow-ups were most common, with the longest follow-ups taking place at 3 years.

### Barriers & facilitators

Barriers to KT were discussed much more frequently than facilitators. Similar to our findings regarding effective intervention components (and consistent with findings from a previous review) [72], the barriers and facilitators reported were more often those found previously in the literature or were based on anecdotal evidence from the current research, rather than the result of systematic inquiry. Often it was unclear how the authors had arrived at the identification of a particular barrier or facilitator.

Two general types of barriers were discussed: 1) implementation barriers (i.e., factors making implementation of the intervention more difficult) and 2) uptake barriers (i.e., factors making uptake, whether in the form of knowledge or behaviour change, more difficult). Uptake barriers were most commonly discussed. Examples included: time constraints and competing demands (e.g., impeding routine IPV screening), lack of privacy (preventing private discussion of IPV/CM) and concerns regarding inadequate or unavailable services/resources for identified victims. As mentioned above, however, these barriers were usually not identified as a result of systematic assessment. Another major barrier discussed in a number of articles highlights the unique nature of IPV/CM – that the content itself is challenging. IPV/CM knowledge can be highly emotional for some, and even hotly debated, as in the case of the efficacy of IPV screening [69]. In particular, articles described barriers such as fears of litigation (in child abuse cases), beliefs regarding professional roles (e.g., believing it isn’t one’s ‘place’ to get involved), inaccurate beliefs about the prevalence of IPV or CM, lack of comfort with IPV or CM subject matter, and the influence of personal experiences of violence (particularly in female-dominated fields such as nursing). These factors can make recipients less receptive to engaging with the KT intervention itself and/or impede motivation or ability to initiate subsequent behaviour change. Several interventions were particularly framed as addressing such barriers, for example interventions to improve provider comfort, including self-efficacy and acceptability, with IPV or CM knowledge e.g., [98, 106, 107]. Others suggested the inclusion of specific components to address the challenges of IPV/CM subject matter such as a hotline [103] or online chat feature [107] to provide support for those participating in online training. The most commonly discussed implementation barriers were the costs involved (especially for complex interventions) and time (i.e., to plan or participate in the intervention). Both have been identified in past research, but, in the articles included in our review, were not generally identified through systematic assessment.

A few facilitators to KT were repeatedly present in the articles. Factors facilitating the implementation of KT interventions were cost-effectiveness and ease of implementation, as is the case with brief workshops or self-led computer or web-based interventions. Another key facilitator, with potential to influence both implementation and uptake, has to do with organization and system-level support. For example, supportive organizational cultures, integration of interventions into settings or “institutionalized approaches” [91], p. 967, and accommodations such as providing relief for staff to attend training were seen as facilitative^b^. As with barriers however, we found, overall, a lack of evidence put forth to support authors’ claims; we interpret them as potentially promising facilitating factors. With regard to barriers and facilitators, there was not enough systematic assessment to do sub-analyses, such as examining whether or not identified barriers and facilitators differed by focus (IPV or CM) or KT recipient.

## Discussion

**Figure 2:**
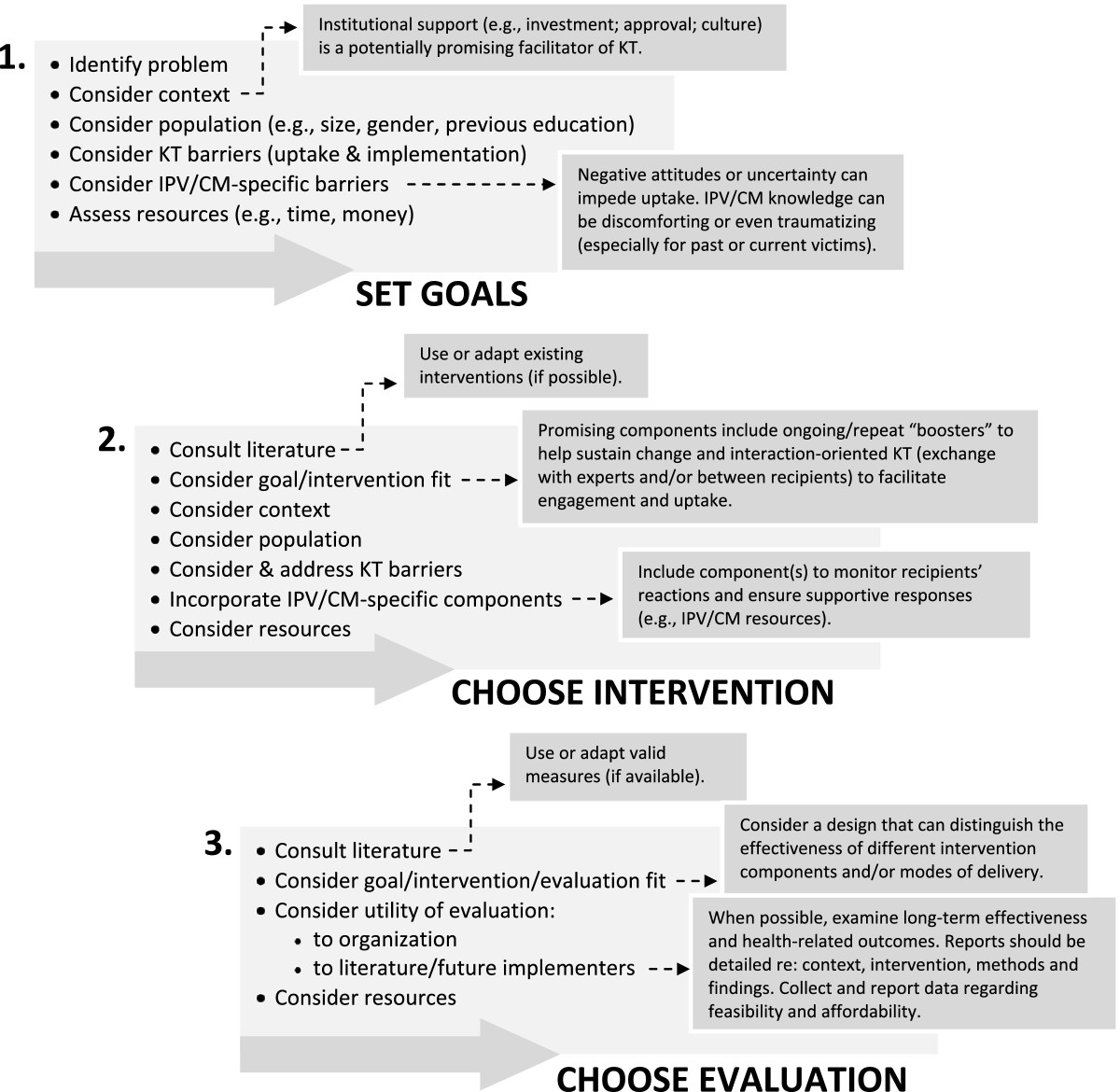
**Proposed Framework for Planning IPV/CM Knowledge Translation Interventions.**

To our knowledge, this review is the most comprehensive synthesis of the IPV and CM KT literature to date, and takes a sufficiently broad definition of KT to provide guidance across a range of potential interventions targeting multiple types of users/audiences when compared to related reviews e.g., [72]. Similar to previous reviews, however, we found multiple gaps in the literature; we review these gaps below according to each research question. Taken together, these gaps make it challenging for future KT implementers to choose modes of delivery, essential components to include, the required level of complexity and frequency of engagement, and, importantly, to know whether a given strategy will actually result in better outcomes for IPV or CM victims. However, to assist in these crucial processes, we provide a guiding framework, described below and presented in Figure 2, regarding key factors and processes found to be promising in this evidence base.

### The extent of IPV and CM-specific KT research

Strides in the last decade have certainly been made. We found 62 articles, including 5 reviews, of sufficient quality to warrant inclusion in this review. There were also many articles reporting on potentially useful interventions that did not meet quality standards, often due to poor research methodology or lack of detail regarding intervention strategies and/or the methods used to evaluate them. Among the 62 articles, we found a great deal of diversity – so much so that we suggest that an underuse of existing KT strategies and measurement tools in the design of KT interventions and their evaluation is a key gap in this literature. We also found almost no examination of health-related outcomes for those exposed to violence. Several authors specifically noted that their research was limited by not knowing how the intervention actually impacted victims. Given the difficulty in conducting this type of research, it is not surprising that this remains a considerable – but important – gap in the literature.

### Effective KT strategies

Consistent with previous reviews [72, 91], we found that many KT strategies to improve IPV/CM knowledge/attitudes were successful, at least in the relative short-term. Studies with long-term follow-up are a research priority. Although behaviour change is ultimately of greater interest, knowledge/attitude change is a crucial part of the change process [108, 109], and given its popularity (and ease of measurement) as an outcome variable, it appears to be recognized as such. It is a welcome finding that complex/multifaceted interventions, which are presumably the most difficult and costly to implement^c^ of all strategies, are not required to successfully influence this proximate outcome; however some evidence, mostly from a systematic review of strategies to increase IPV screening and identification rates [87], suggests that these may be the most effective. Influencing behaviour (and then assessing whether behaviour change by practitioners facilitates important outcomes in those exposed to violence) is understandably more difficult, as the mixed findings of multiple reviews indicate. Nevertheless, many interventions were found to be successful in changing behaviour or behavioural intention. Given the large number of articles in our sample focused on the detection and documentation of CM or IPV cases, however, we recommend cautious interpretation of these findings. The extent to which findings from these studies can be generalized to other types of behavioural outcomes is unclear; more research is needed to determine the effectiveness of various KT strategies to change other types of behaviour related to IPV and CM. Furthermore, although we identified several components of interventions that are promising, such as ongoing or ‘booster’ training sessions, opportunities for exchange, and components that make organizational cultures more conducive to KT, methodological limitations prevent us from drawing strong conclusions. Similarly, with regard to the complex/multifaceted interventions in their review, Larrivée et al. [72], p. 2387 concluded that “it is impossible to isolate the effect of one strategy [i.e., component] from that of another.” Identifying these ‘active ingredients’ is important, however, particularly in the case of complex/multifaceted interventions, where trimming unnecessary components could save considerable time and money, known barriers to implementation. A strong understanding of effective/ineffective KT intervention components, and modes of delivery, based on systematic inquiry, remains a key gap in the literature.

### Barriers & facilitators

Overall, we found that authors’ conclusions regarding barriers and facilitators were often not grounded in systematic inquiry – i.e., the conclusions were sometimes not warranted given the findings of the study. This is especially problematic given that researchers or other ‘implementers’ may be looking to this kind of research for guidance. Moreover, it has been suggested that KT interventions based on assessments of barriers and facilitators are more likely to be effective [110], indicating that this is an important first step in the knowledge-to-action process. Nevertheless, a key theme arising from the data was the unique, often challenging, nature of IPV and CM knowledge and the potential for this to act as a barrier to KT implementation and uptake.

### Broader KT literature

Many of our findings appear to align well with findings from other areas of health care KT, suggesting that while context is critical in the particulars of how knowledge is taken-up by different actors in different settings [56], including IPV/CM knowledge, some aspects may transfer from context to context. For example, the finding that complex/multifaceted interventions are particularly effective is consistent with a review of systematic reviews on interventions to improve quality of care [111], and with arguments that complex solutions are needed to solve complex public health problems situated in complex systems e.g., [112]. In the same review of reviews, Grimshaw et al. [111] also highlighted the unresolved issue of disentangling which components of multifaceted interventions are effective. The role of interaction-oriented KT interventions – those that integrate exchange between producers and knowledge users, but also those that involve interaction amongst knowledge users themselves – is understudied, though some studies show these strategies to be potentially promising e.g., [37, 113]. Unlike Bloom’s [114] review of systematic reviews on continuing medical education strategies to improve physician care and patient outcomes, we did not find that passive strategies were the most commonly used. It is unclear whether this reflects conclusions regarding the general ineffectiveness of such strategies [114], or a general trend away from such strategies in more recent years. Overall, it is likely that decisions regarding IPV/CM KT strategies should be based on the best available evidence from within and outside the IPV/CM domain.

### Consideration of health inequities

Discussions of violence and violence exposure are increasingly intermixed with understanding that these experiences are strongly related to health inequities, social determinants of health and social justice [115]. This is true at the level of individual risk factors for IPV and CM exposure, as well as the interaction of family, community and structural factors, as outlined in the World Health Organization’s (WHO) ecological framework for understanding violence [2]. Health equity means that equal opportunities for health are experienced by those who receive health services [115, 116]. This focus is reflected in the United Nations Millennium Development Goals, for example, which call for monitoring social determinants of health to mitigate disadvantages due to socially determined positions, such as poverty or race. The WHO outlines strategies to address embedded, upstream sociopolitical structures [117, 118]. Downstream strategies relevant to implementation science and the way that IPV/CM knowledge is integrated into practice can include, for example, ensuring reasonable access to services through the provision of material resources (e.g., free babysitting or bus tickets), or tailoring IPV/CM services to priority populations (e.g., cultural groups, bi/gay/lesbian groups). Tools, such as the USAID Checklist for Health Equity Programming [119] are available for program planners working in this area. Health inequities are multifactorial, requiring both upstream and downstream approaches to ensure that those affected by IPV/CM are able to achieve their full health potential.

### A guiding framework

Despite the gaps outlined above, we find the evidence base in this area to be of sufficient size and quality to be useful. As such, based on our summary and synthesis of this literature, as well as key findings from the broader KT literature, we propose a guiding framework to assist those wishing to implement and evaluate KT strategies in this area (e.g., program planners; see Figure 2). Our framework provides suggestions for key considerations at each of three potentially iterative decision-making stages: 1) setting goals, 2) choosing the intervention, and 3) choosing the evaluation. It incorporates, where possible, specific recommended strategies for IPV/CM KT. Several suggested considerations appear in our framework under more than one stage in the process. For example, our recommendation to ‘consider context’ and ‘consider population’ appears in the first two stages of the framework and highlights the importance that we, and many others e.g., [57], place on contextualized approaches to KT. We suggested in our introduction that IPV/CM KT is, by virtue of its complexity, prevalence (including among potential recipients of KT interventions) and psycho-social nature, substantively different than many topics that have been studied from a health KT perspective. In addition, relevant research may come from a variety of settings – in fact up to half of the articles reported intervention recipients who were not health care providers, but included police, educators and others who interact with victims of violence. In fewer cases was the KT implemented specifically or directly in a community-based setting. While scholars have suggested that implementation studies would benefit from greater responsiveness to the needs and preferences of diverse populations (e.g., cultural groups), we did not find evidence of such considerations in our review [120], and this remains a research priority. Nevertheless, to ensure KT interventions in this area are culturally sensitive and safe, the cultural background(s) of recipients may be an important factor to consider. Other ‘contextual’ issues such as whether or not institutionalized support exists for the KT, how much IPV/CM education recipients already have, recipient gender, and how much time recipients have to participate in KT initiatives should also be considered. As Wathen et al. [37], p. 14 conclude, “[o]ne-size-fits-all approaches to KTE do not address the complexities and particularities of specific contexts, nor the interaction of contextual factors with ‘evidence’.”

While considering context is crucial, a number of the interventions we reviewed did show promise, and a first step is that would-be implementers consult the literature when considering potential KT strategies and their evaluation. Existing interventions can be used either in part, in an adapted form, or in some cases in their entirety, depending on the available resources, goals of the initiative, and suitability for the context. Similarly, when possible and appropriate, we recommend the use (or, at minimum, the consultation or adaptation) of established measurement tools and evaluation approaches that have been shown to be effective or promising. This evidence-based approach will reduce heterogeneity in the literature, and facilitate more effective KT implementation.

KT in the area of IPV/CM presents a unique challenge because of the emotional, and sometimes contested, nature of the subject matter and of the emerging evidence in the field. For this reason, in the first stage of our framework, we recommend that those planning an intervention take special care to consider potential IPV/CM-specific barriers. For example, given studies indicating both the lifetime prevalence of CM and IPV generally, and among healthcare providers specifically [121], it should be assumed that some recipients will have personal experience with violence and we therefore recommend that a trauma and violence informed practice lens be used in delivering KT interventions [122, 123]. In the second stage, we encourage implementers to incorporate appropriate ways of monitoring and addressing recipients’ responses to IPV/CM knowledge into the intervention (e.g., making supportive resources available).

Another key aspect of our proposed framework, present in the third stage, highlights implementers’ responsibility to maximize the utility of their evaluation efforts. Our recommendations include: examining long-term effectiveness and health-related outcomes, employing evaluation approaches capable of distinguishing effective/ineffective intervention components/modes, and reporting thorough accounts of the research context, intervention, methods, implementation process and findings, including feasibility and affordability.

### Limitations

Our findings may be limited in several ways. First, they may be subject to publication bias because of the exclusion of grey literature (e.g., government reports, dissertations, etc.). Second, some relevant research, particularly if framed differently or lacking description of the KT component of an intervention, may not have been caught by our searches. Finally, although we view our broad conceptualization of KT (especially the inclusion of continuing education interventions) as a strength^d^, and ideal for capturing the full range of ways that knowledge about IPV and CM can be communicated to various audiences, some may view it as a weakness; much of the research included in our review may not fit the prototypical form of KT described in the KT literature e.g., [49]. In fact, only a small percentage of included articles used KT terms to describe the intervention. Similarly, articles specifying that the content of the intervention (i.e., the ‘K’ in ‘KT’) was based on research evidence were in the minority (less than 25%) and were often difficult to clearly discern; therefore, including these as a necessary component of KT would also have drastically restricted our sample. These findings highlight the ‘indistinct terrain’ of KT and emphasize the need for clearer definitions and distinctions among the various forms of KT. Our conceptualization of ‘exchange’ is also more broad, and most examples of it in our sample do not reflect the in-depth process of linkage and interaction between researchers and knowledge users promoted by KT researchers e.g., [37, 59]. Nevertheless, our review captures the scope of IPV/CM KT research, such as it is.

## Conclusion

Although further research is needed, especially in community-based settings [53], we are encouraged by the number of studies conducted in this area and take it as an indication that the importance of improving methods to identify, address, and prevent IPV and CM is recognized by many. Our guiding framework provides a clear starting place for those wishing to implement and evaluate KT in this area. We hope that the development of partnerships and increased communication amongst interested parties (violence researchers, advocates, program planners, etc.), in combination with thorough reporting of evaluation efforts, may result in a more useful literature highlighting effective interventions and, ultimately, better outcomes for victims of violence. We also encourage those working in the field to consider the intersection of CM and IPV research and KT efforts in several respects. First, children’s exposure to IPV is a recognized form of maltreatment, and strategies are required to generate, translate and use evidence to prevent and respond to this form of violence in the home – both for the abused caregiver, as well as to reduce the negative consequences for the exposed child(ren) [31–33]. Second, these forms of violence are often intergenerational (e.g., victims of CM are more likely to grow up to be victims and/or perpetrators of all forms of interpersonal violence), and the consequences of exposure to violence occur across the lifespan, especially in terms of mental health outcomes [124–126]. These more nuanced understandings of IPV and CM as phenomena, combined with appropriate use of a health equity lens and embedded in KT strategies for educating a broad range of health and social service actors, will be of greatest benefit to those experiencing violence and its consequences.

## Endnotes

^a^Insufficient intervention detail is relatively rare in our sample given that this feature tended to be present in articles of overall poor quality which were excluded from this review.

^b^It should be noted that although normally discussed separately, each barrier can be framed as a facilitator, and vice versa – for example, organizational support facilitates KT, but a lack of organizational support may impede it.

^c^Reports of feasibility and affordability were rare in our sample.

^d^In a systematic review of RCTs of IPV educational interventions for health care providers published following the present analysis, Zaher et al. [127] noted similar patterns for identification and referral, supporting our decision to include such research.

## Electronic supplementary material

Additional file 1: Database Search Terms.(DOCX 13 KB)

Additional file 2: Reference List of All 62 Articles Included in Review.(DOCX 47 KB)

Additional file 3: Summary of All 62 Articles Included in Review.(DOCX 31 KB)

Additional file 4: Quality Appraisal for 52 Excluded Articles.(DOCX 27 KB)

Below are the links to the authors’ original submitted files for images.

Authors’ original file for figure 1

Authors’ original file for figure 2

## Abbreviations

CM: Child maltreatment
EBM: Evidence-based medicine
IPV: Intimate partner violence
KT: Knowledge translation
NGO: Non-governmental organization
RCT: Randomized controlled trial.
