# Supplementary material for: Strategies to promote uptake and use of intimate partner violence and child maltreatment knowledge: an integrative review
Source: BMC Public Health. 2014 Aug 21;14:862. doi: 10.1186/1471-2458-14-862 (PMC4152574; doi:10.1186/1471-2458-14-862)
Supplement: Supplementary file 1 — Additional file 1: Database Search Terms.(DOCX 13 KB) [file 12889_2014_6991_MOESM1_ESM.docx]

Additional File 1: Database Search Terms

| **Controlled Vocabulary** | |
| --- | --- |
| **Embase (Emtree)** |  |
| KT | information dissemination, evidence based medicine, evidence based practice |
| CE | continuing education, in service training |
| IPV/CM | domestic violence, exp battered woman, exp child abuse, exp family violence, exp partner violence, exp child neglect |
| **CINAHL** |  |
| KT | exp knowledge, “medical practice, evidence-based”, “medical practice, research-based” |
| CE | “education, continuing”, staff development |
| IPV/CM | domestic violence, intimate partner violence, exp child abuse, dating violence |
| **ERIC** |  |
| KT | information dissemination, research utilization, information utilization, educational indicators, program effectiveness |
| CE | exp continuing education, exp mandatory continuing education, exp professional continuing education, exp continuing education centers, exp continuing education units, exp inservice education, exp inservice teacher education, orientation, staff orientation |
| IPV/CM | family violence, child abuse, child neglect |
| **PsycInfo** |  |
| KT | knowledge transfer, information dissemination |
| CE | continuing education, inservice training |
| IPV/CM | exp child abuse, exp intimate partner violence, exp battered females, exp partner abuse |
| **Medline (MeSH)** |  |
| KT | knowledge, “health knowledge, attitudes, practice”, evidence-based medicine, diffusion of innovation, information dissemination |
| CE | exp “education, continuing”, exp inservice training |
| IPV/CM | domestic violence, child abuse, spouse abuse, battered women |
| **Soc. Abstracts** |  |
| KT | diffusion, knowledge utilization, information dissemination, evidence-based practice |
| CE | adult education, work orientations, professional orientations |
| IPV/CM | battered women, spouse abuse, child neglect, family violence, partner abuse, child neglect |
| **Keywords*** | |
| KT | guideline implementation, know-do gap, knowledge-to-action, dissemination, (knowledge, research, or information) with (translat*, transfer*, utiliz*, utilis*, mobiliz*, mobilis*, implementation, cycle, broker*, adoption, uptake, or diffusion) |
| CE | continu* with (educat*, train*, school*, or professional* develop*), (inservice, in service, or in-service) with (train*, educat*, or school*) |
| IPV/CM | (violence, abus*, or batter*) with (partner, domestic, spous*, family, conjugal, wife, wives, husband*, or women), child* with (maltreatment, neglect, abuse, or batter*) |

Note: ‘exp’ denotes that a controlled term has been ‘exploded’ (i.e., controlled terms below it in hierarchy were also searched). KT = knowledge translation, CE = continuing education, IPV/CM = intimate partner violence/child maltreatment

*Keywords were used in combination with controlled vocabulary and the same keywords were used for all databases.
