# Supplementary material for: Strategies to promote uptake and use of intimate partner violence and child maltreatment knowledge: an integrative review
Source: BMC Public Health. 2014 Aug 21;14:862. doi: 10.1186/1471-2458-14-862 (PMC4152574; doi:10.1186/1471-2458-14-862)
Supplement: Supplementary file 2 — Additional file 2: Reference List of All 62 Articles Included in Review.(DOCX 47 KB) [file 12889_2014_6991_MOESM2_ESM.docx]

Additional File 2: Reference List of All 62 Articles Included in Review

| 1 | Hazzard A: **Training teachers to identify and intervene with abused children.** *J Clin Child Psychol* 1984, **13:**288-293. |
| --- | --- |
| 2 | Lamb ME, Sternberg KJ, Orbach Y, Hershkowitz I, Horowitz D, Esplin P: **The effects of intensive training and ongoing supervision on the quality of investigative interviews with alleged sex abuse victims.** *Appl Dev Sci* 2000, **6:**114-125. |
| 3 | Warburton AL, Hanif B, Coulthard P: **Changes in the levels of knowledge and attitudes of dental hospital staff about domestic violence following attendance at an awareness raising seminar.** *Brit Dent J* 2006, **201:**653-659. |
| 4 | Agirtan CA, Akar T, Akbas S, Akdur R, Aydin C, Aytar G, Ayyıldız S, Baskan S, Belgemen T, Bezirci O, Beyazova U, Beyaztas FY, Buken B, Buken E, Camurdan AD, Can D, Canbaz S, Cantürk G, Ceyhan M, Coskun A, Celik A, Cetin FC, Coskun AG, Dagcinar A, Dallar Y, Demirel B, Demirogullari B, Derman O, Dilli D, Ersahin Y: **Establishment of interdisciplinary child protection teams in Turkey 2002-2006: Identifying the strongest link can make a difference!** *Child Abuse Neglect* 2009, **33:**247-255. |
| 5 | Cyr M, Lamb ME: **Assessing the effectiveness of the NICHD investigative interview protocol when interviewing French-speaking alleged victims of child sexual abuse in Quebec.** *Child Abuse Neglect* 2009, **33:**257-268. |
| 6 | Lia-Hoagberg B, Schaffer M, Strohschein S: **Public health nursing practice guidelines: An evaluation of dissemination and use.** *Public Health Nurs* 1999, **16:**397-404. |
| 7 | O’Campo P, Kirst M, Tsamis C, Chambers C, Ahmad F: **Implementing successful intimate partner violence screening programs in health care settings: Evidence generated from a realist-informed systematic review.** *Soc Sci Med* 2011, **72:**855-866. |
| 8 | Louwers E, Affourtit MJ, Moll HA, de Koning HJ, Korfage IJ: **Screening for child abuse at emergency departments: A systematic review.** *Arch Dis Child* 2010, **9*5*:**214-218. |
| 9 | Boursnell M, Prosser S: **Increasing identification of domestic violence in emergency departments: A collaborative contribution to increasing the quality of practice of emergency nurses.** *Contemp Nurse* 2010, **35:**35-46. |
| 10 | Heyman RE, Smith Slep AM: **Reliability of family maltreatment diagnostic criteria: 41 site dissemination field trial.** *J Fam Psychol* 2009, **23:**905-910. |
| 11 | Hsieh NK, Herzig K, Gansky SA, Danley D, Gerbert B: **Changing dentists’ knowledge, attitudes and behavior regarding domestic violence through an interactive multimedia tutorial.** *J Am Dent Assoc* 2006, **137:**596-603. |
| 12 | Rischke AE, Roberts KP, Price HL: **Using spaced learning principles to translate knowledge into behavior: Evidence from investigative interviews of alleged child abuse victims.** *J Police Crim Psych* 2011, **26:**58-67. |
| 13 | Wathen CN, Sibbald SL, Jack SM, Macmillan HL: **Talk, trust and time: A longitudinal study evaluating knowledge translation and exchange processes for research on violence against women.** *Implement Sci* 2011, **6:**102. |
| 14 | Allert CS, Chalkley C, Whitney JR, Librett A: **Domestic violence: Efficacy of health provider training in Utah.** *Prehosp Disaster Med* 1997, **12:**52-56. |
| 15 | Aved BM, Meyers L, Burmas EL: **Challenging dentistry to recognize and respond to family violence.** *J Calif Dent Assoc* 2007, **35:**555-563. |
| 16 | Barber-Madden R: **Training day care program personnel in handling child abuse cases: Intervention and prevention outcomes.** *Child Abuse Neglect* 1983, **7:**25-32. |
| 17 | Berger RP, Bogen D, Dulani T, Broussard E: **Implementation of a program to teach pediatric residents and faculty about domestic violence.** *Arch Pediat Adol Med* 2002, **156:**804-810. |
| 18 | Botash AS, Galloway AE, Booth T, Ploutz-Snyder R, Hoffman-Rosenfeld J, Cahill L: **Continuing medical education in child sexual abuse: Cognitive gains but not expertise.** *Arch Pediat Adol Med* 2005, **159:**561-566. |
| 19 | Campbell JC, Coben JH, McLoughlin E, Dearwater S, Nah G, Glass N, Lee D, Durborow N: **An evaluation of a system-change training model to improve emergency department response to battered women.** *Acad Emerg Med* 2001, **8:**131-138. |
| 20 | Chaffin M, Kelleher K, Harber G, Harper J: **Impact of substance abuse and child maltreatment training on service utilization in a rural setting.** *J Child Family Stud* 1994, **3:**379-387. |
| 21 | Cross W, Cerulli C: **The impact of continuing education training on law guardian knowledge, efficacy, and practice behaviors.** *Fam Court Rev* 2007, **45:**92-102. |
| 22 | Darby JA: **Evaluating course evaluations: The need to establish what is being measured.** *Assess Eval Higher Educ* 2007, **32:**441-455. |
| 23 | Davidson LL, Grisso JA, Garcia-Moreno C, Garcia J, King VJ, Marchant S: **Training programs for healthcare professionals in domestic violence.** *J Women Health Gen-B* 2001, **10:**953-969. |
| 24 | Davila YR: **Increasing nurses' knowledge and skills for enhanced response to intimate partner violence.** *J Contin Educ Nurs* 2006, **37:**171-177. |
| 25 | Dubowitz H, Lane WG, Semiatin JN, Magder LS, Venepally M, Jans M: **The safe environment for every kid model: Impact on pediatric primary care professionals.** *J Pediatr* 2011, **127:**962-970. |
| 26 | Harris JC, Bradbury J, Porritt J, Nilchian F, Franklin CD: **NHS dental professionals' evaluation of a child protection learning resource.** *Brit Dent J* 2011, **210:**75-79. |
| 27 | Harris JM, Kutob RM, Surprenant ZJ, Maiuro RD, Delate TA: **Can Internet-based education improve physician confidence in dealing with domestic violence?** *Fam Med* 2002, **34:**287-292. |
| 28 | Hawkins R, McCallum C: **Mandatory notification training for suspected child abuse and neglect in south australian schools.** *Child Abuse Neglect* 2001, **25:**1603-1625. |
| 29 | Hibbard RA, Serwint J, Connolly M: **Educational program on evaluation of alleged sexual abuse victims.** *Child Abuse Neglect* 1987, **11:**513-519. |
| 30 | Jones JG, Garrett J, Worthington T: **A videotape series for teaching physicians to evaluate sexually abused children.** *J Child Sex Abuse* 2004, **13:**87-97. |
| 31 | Khan AN, Rubin DH, Winnik G: **Evaluation of the mandatory child abuse course for physicians: Do we need to repeat it?** *J Public Health* 2005, **119:**626-631. |
| 32 | Kleemeier C, Webb C, Hazzard A: **Child sexual abuse prevention: Evaluation of a teacher training model.** *Child Abuse Neglect* 1988, **12:**555-561. |
| 33 | McCosker H, Madl R, Harris M, Anderson D, Mannion J: **Evaluation of a self-paced education package on violence against women for rural community-based health workers.** *Aust J Rural Health* 1999, **7:**5-12. |
| 34 | McGrath P, Cappelli M, Wiseman D, Khalil N, Allan B: **Teacher awareness program on child abuse: A randomized controlled trial.** *Child Abuse Neglect* 1987, **11:**125-132. |
| 35 | Newton AS, Zou B, Hamm MP, Curran J, Gupta S, Dumonceaux C, Lewis M: **Improving child protection in the emergency department: A systematic review of professional interventions for health care providers.** *Acad Emerg Med* 2010, **17:**117-125. |
| 36 | Nicolaidis C, Curry M, Gerrity M: **Measuring the impact of the voices of survivors program on health care workers' attitudes toward survivors of intimate partner violence.** *J Gen Intern Med* 2005, **20:**731-737. |
| 37 | Paluzzi P, Gaffikin L, Nanda J: **The american college of nurse-midwives' domestic violence education project: Evaluation and results.** *J Midwifery Womens Health* 2000, **45:**384-391. |
| 38 | Protheroe L, Green J, Spiby H: **An interview study of the impact of domestic violence training on midwives.** *J Midwifery* 2004, **20:**94-103. |
| 39 | Salmon D, Murphy S, Baird K, Price S: **An evaluation of the effectiveness of an educational programme promoting the introduction of routine antenatal enquiry for domestic violence.** *Midwifery* 2006, **22:**6-14. |
| 40 | Saunders DG, Holter MC, Pahl LC, Tolman RM, Kenna CE: **TANF workers’ responses to battered women and the impact of brief worker training: What survivors report.** *Violence Against Women* 2005, **11:**227-254. |
| 41 | Schoening AM, Greenwood JL, McNichols JA, Heermann JA, Agrawal S: **Effect of an intimate partner violence educational program on the attitudes of nurses.** *J Obstet Gynecol Neonatal Nurs* 2004, **33:**572-579. |
| 42 | Shefet D, Dascal-Weichhendler H, Rubin O, Pessach N, Itzik D, Benita S, Ziv A: **Domestic violence: A national simulation-based educational program to improve physicians' knowledge, skills and detection rates.** *Med Teach* 2007, **29:**133-138. |
| 43 | Short LM, Surprenant ZJ, Harris JM: **A community-based trial of an online intimate partner violence CME program.** *Am J Prev Med* 2006, **30:**181-185. |
| 44 | Smeekens AE, Broekhuijsen-van Henten DM, Sittig JS, Russel IM, ten Cate OT, Turner NM, van de Putte EM: **Successful e-learning programme on the detection of child abuse in emergency departments: A randomised controlled trial.** *Arch Dis Child* 2011, **96:**330-334. |
| 45 | Socolar RR, Raines B, Chen-Mok M, Runyan DK, Green C, Paterno S: **Intervention to improve physician documentation and knowledge of child sexual abuse: A randomized, controlled trial.** *J Pediatr* 1998, **101:**817-824. |
| 46 | Sullivan R, Clancy T: **An experimental evaluation of interdisciplinary training in intervention with sexually abused adolescents.** *Health Soc Work* 1990, **15:**207-214. |
| 47 | Walker SL, Smith DJ: **“Children at risk”: Development, implementation, and effectiveness of a school-based violence intervention and prevention program.** *J Prev Interv Community* 2009, **37:**316-325. |
| 48 | Young HL, Mancuso AF, Faherty E, Dorman SA, Umbrell JR: **Helping child victims of family violence through school personnel: An evaluation of a training program.** *J* *Aggress Maltreat Trauma* 2008, **16:**144-163. |
| 49 | Zachary MJ, Schechter CB, Kaplan ML, Mulvihill MN: **Provider evaluation of a multifaceted system of care to improve recognition and management of pregnant women experiencing domestic violence.** *Womens Health Issues* 2002, **12:**5-15. |
| 50 | Cerezo A, Pons-Salvador G: **Improving child maltreatment detection systems: A large scale case study involving health, social services and school professionals.** *Child Abuse Neglect* 2004, **28:**53-69. |
| 51 | Dresser MG, Short L, Wedemeyer L, Bredow VL, Sacks R, Larson K, Levy J, Silver LD: **Public health detailing of primary care providers: New york city's experience, 2003 -2010.** *Am J Prev Med* 2012, **102:**342-352. |
| 52 | Larrivée M, Hamelin-Brabant L, Lessard G: **Knowledge translation in the field of violence against women and children: An assessment of the state of knowledge.** *Child Youth Serv Rev* 2012, **34:**2381-2391. |
| 53 | Paranal R, Washington Thomas K, Derrick C: **Utilizing online training for child sexual abuse prevention: Benefits and limitations.** *J Child Sex Abuse* 2012, **21:**507-520. |
| 54 | Rheingold AA, Zajac K, Patton M: **Feasibility and acceptability of a child sexual abuse prevention program for childcare professionals: Comparison of a web-based and in-person training.** *J Child Sex Abuse* 2012, **21:**422-436. |
| 55 | Whitaker DJ, Ryan KA, Wild RC, Self-Brown S, Lutzker JR, Shanley JR, Edwards AM, McFry EA, Moseley CN, Hodges AE: **Initial implementation indicators from a statewide rollout of safecare within a child welfare system.** *Child Maltreatment* 2012, **17:**96-101. |
| 56 | Janssen PA, Holt VL, Sugg NK: **Introducing domestic violence assessment in a postpartum clinical setting.** *Matern Child Health J* 2002, **6:**195-203. |
| 57 | Thompson RS, Rivara FP, Thompson DC, Barlow WE, Sugg NK, Maiuro RD, Rubanowice DM: **Identification and management of domestic violence: A randomized trial.** *Am J Prev Med* 2000, **19:**253-263. |
| 58 | Bonds DE, Ellis SD, Weeks E, Palla SL, Lichstein P: **A practice-centered intervention to increase screening for domestic violence in primary care practices.** *BMC Fam Pract* 2006, **7:**1-8. |
| 59 | Feder G, Davies RA, Baird K, Dunne D, Eldridge S, Griffiths C, Gregory A, Howell A, Johnson M, Ramsay J, Rutterford C, Sharp D: **Identification and referral to improve safety (IRIS) of women experiencing domestic violence with a primary care training and support programme: A cluster randomised controlled trial.** *Lancet* 2011, **378:**1788-1795. |
| 60 | Knapp JF, Dowd MD, Kennedy CS, Stallbaumer-Rouyer J, Henderson DP: **Evaluation of a curriculum for intimate partner violence screening in a pediatric emergency department.** *J Pediatr* 2006, **117:**110-116. |
| 61 | Lo Fo Wong S, Wester F, Mol SS, Lagro-Janssen TL: **Increased awareness of intimate partner abuse after training: A randomised controlled trial.** *Brit J Gen Pract* 2006, **56:**249-257. |
| 62 | Olson L, Anctil C, Fullerton L, Brillman J, Arbuckle J, Sklar D: **Increasing emergency physician recognition of domestic violence.** *Ann Emerg Med* 1996, **27:**741-746. |
