# Supplementary material for: Strategies to promote uptake and use of intimate partner violence and child maltreatment knowledge: an integrative review
Source: BMC Public Health. 2014 Aug 21;14:862. doi: 10.1186/1471-2458-14-862 (PMC4152574; doi:10.1186/1471-2458-14-862)
Supplement: Supplementary file 3 — Additional file 3: Summary of All 62 Articles Included in Review.(DOCX 31 KB) [file 12889_2014_6991_MOESM3_ESM.docx]

Additional File 3: Summary of All 62 Articles Included in Review

| **First Author, Year (Country)** | **Study Design (focus)** | **Intervention Type/Details** | **Sample/KT ‘recipients’** | **Key Strengths (S) & Weaknesses (W)** | **Outcomes/Key Findings** |
| --- | --- | --- | --- | --- | --- |
| Agirtan, 2009 (Turkey) | Other (multiple measurement points, depends on outcome variable; CM) | Unclear/unknown: needs-based training in 3 cities over 2 weeks annually, 10 train-the-trainer sessions, 8 symposia over 5 years | Varied (e.g., law enforcement, social workers, nurses) | S: good description of research context, novel intervention and outcomes  W: lacking detail regarding intervention and data collection, high loss to follow-up | - increased number of multidisciplinary teams (MDTs) and lectures/conferences held by MDTs  - increased number of abuse cases assessed by MDTs |
| Allert, 1997 (US) | Pre-post (immediate) with 3-month follow-up (IPV) | Multimode with exchange: 1.5 hour in-service training session (didactic, video, discussion) | Health practitioners (e.g., paramedics, ED staff) | S: good sample size  W: outcome measures limited | - improved referral and legal knowledge, confidence in asking about IPV  - intervention generally not effective in changing attitudes or self-reported screening behaviour |
| Aved, 2007 (US) | Other (post-only; IPV and CM) | Passive/didactic: 4-hour seminar, train-the-trainer workshops (length unknown) | Varied (e.g., dentists, hygienists, nurses) | S: good sample size, varied outcome measures  W: all outcomes self-report | - Ps perceived increases in knowledge and confidence in recognizing violence pre- to post-training  - just over half the trainers had implemented a training session |
| Barber-Madden, 1983 (US) | Quasi-experiment (CM) | Multimode with exchange: 6 3-hour workshops (monthly; assignments to complete, discussion, specific activities tailored according to needs of site) | Varied (child care workers, program directors, social service coordinators) | S: good description of research context and attempt to measure behavioural outcomes  W: lacking sufficient detail regarding intervention and analyses | - increased referrals, likelihood of developing written CM policy, involvement in prevention activities in year following intervention  - treatment and control groups did not differ on behavioural outcomes (e.g., reporting) |
| Berger, 2002 (US) | Pre-post (IPV and CM) | Complex/multifaceted: 30-minute didactic session, 90-minute session (didactic, video, role-play), posters, tailored content | Health practitioners (e.g., physicians, nurses) | S: clear rationale for intervention design, measurement tools, good methodological detail  W: small sample size, self-report only | - knowledge high at pre-test, no change post-test  - improvements in self-reported IPV screening practices, identification, and awareness of resources |
| Bonds, 2006 (US) | Pre-post (IPV) | Complex/multifaceted: customized intervention with multiple training sessions, available ongoing support, designated IPV resource persons | Health practitioners (e.g., physicians, nurses) | S: behavioural outcome, detailed reporting of methods, intervention, etc.  W: lacking good description of research setting | - Increased IPV screening rates |
| Botash, 2005 (US) | Pre-post (CM) | Multimode without exchange: case-based self-study including workbook and videotaped instruction | Health practitioners (e.g., physicians, nurses) | S: good intervention description  W: insufficient detail regarding research setting and measures | - improved knowledge (e.g., physical examinations, anatomy)  - post-test essays were generally poor with Ps no properly reassuring parents, interpreting physical exams, or indicating proper legal implications |
| Boursnell, 2010 (Australia) | Pre-post with 6-month follow-up (IPV & CM; mixed method) | Other: 1-hour training (including video), screening pathway displayed, research staff site visits | Health practitioners (nursing staff) | S: intervention, method, and measures well-described, with rationales  W: some loss to follow-up | - increased awareness of responsibilities and policy, confidence in ability to identify and refer  - file audit revealed increased asking about children in IPV presentations |
| Campbell, 2001 (US) | Experiment/RCT with pre-post (IPV; plus 9-12 and 18-24 month follow-up; mixed method) | Complex/multifaceted: 2-day session (didactic, role-play, team action planning), team-initiated/tailored component, researcher support | Varied (e.g., physicians, social workers) | S: strong study design  W: some methodological details lacking | - improved knowledge, attitudes and ED culture in treatment group compared to control  - no group difference in IPV identification |
| Cerezo, 2004 (Spain) | Quasi-experiment with pre-post (CM) | Complex/multifaceted: 16 to 20-hour courses over 2 or 3 days, support and consultation from Local Coordination Team, creation of child protection services referral form | Varied (e.g., nurses, social workers, police) | S: detailed description of research setting, methods and analyses, strong design  W: contamination effect due to media coverage | - Increased detection rate in child protection cases, some differences by region and point of measurement  - Appeared to be linear relationship between number of professionals trained and number of detected cases |
| Chaffin, 1994 (US) | Quasi-experiment with pre-post (CM) | Other: 6-hour didactic/passive training, selected Ps attended additional 8-day training (1 day per month; role-play, case consultation, readings etc.) | Varied (e.g., child protection workers, social workers, nurses) | S: strong study design  W: unknown if regional effects due to referrals made by Ps receiving training, some methodological details lacking | -increase in substance abuse service utilization in treatment region compared to control  - increased knowledge in treatment group at post-test |
| Cross, 2007 (US) | Other (quasi-experiment with 2 post-tests; CM) | Passive/didactic: 1 day ‘conference’ | Other (law guardians/attorneys) | S: good methodological detail  W: low internal reliability for some subscales of outcome measure | -no difference between treatment and control groups on knowledge, but training improved feelings of efficacy and intentions to carry out new practice behaviours |
| Cyr, 2009 (Canada) | Pre-post (CM) | Complex/multifaceted: week-long session (instruction on/practice with interview protocol), continual written feedback on all conducted interviews throughout study period | Varied (e.g., police officers, social workers) | S: good methodological detail  W: description of research setting and questions somewhat lacking | -higher quality forensic interviews conducted at post-test |
| Darby, 2007 (UK) | Quasi-experiment (CM) | Multimode without exchange: 3 1-day sessions held within a few weeks (presentations, groups work, individual activities) | Teachers/educators | S: strong study design, good sample size  W: some detail regarding intervention and behavioural measures lacking | - compared to controls, Ps who took course reported greater use of course materials, but were not more likely to make referrals  - course had positive effect on Ps’ knowledge/attitudes regarding abuse and their role in child protection |
| Davidson, 2001 (UK) | Systematic review (IPV) | Varied (35 papers reviewed) | Health practitioners (e.g., physicians) | S: inclusion criteria and screening process clearly described  W: methodological detail regarding quality appraisal lacking | - Training healthcare providers to screen/ counsel women can increase detection rates  - long-term effectiveness is unknown  - deficiencies in reporting of details of training content, inadequate and varied outcome measures make cross-study comparisons difficult  - few rigorously designed evaluations |
| Davila, 2006 (US) | Pre-post (IPV) | Multimode with exchange: 1 3.5-hour session (lecture, instructive video, panel presentation, question/answer discussion periods) | Health practitioners (e.g., nurse practitioners) | S: good description of intervention, use of established measurement tool  W: high loss to follow-up | - no difference in knowledge post training, but significant improvement in self-reported assessment/ intervention skills |
| Dresser, 2012 (US) | Pre-post (IPV and CM) | Complex/multifaceted: repeat, personal visits to office teams (provide information, support) | Health practitioners (e.g., physicians) | S: good sample size, novel intervention  W: some methodological detail lacking (e.g., regarding loss to follow-up, analyses, question coding) | - Increased routine IPV screening and likelihood of hotline referral and scheduling follow-up  - No improvement in providers reporting child abuse cases or attending extra training, increased likelihood of calling state register |
| Dubowitz, 2011 (US) | Experiment/RCT with pre-post (6, 18, 24, 36 months; CM) | Complex/multifaceted: 4-hour training sessions every 6 months, parent handouts, periodic newsletter, available social worker for support | Health practitioners (e.g., pediatricians, nurse practitioners) | S: strong design and analysis  W: some Ps completed only 1 follow-up | - Increased overall comfort, competence, attitudes, and behaviour (e.g., IPV screening) concerning CM risk factors |
| Feder, 2011 (UK) | Experiment/RCT with pre-post (IPV) | Complex/Multifaceted: two 2-hour training sessions, champion attended 8-hour training, posters, chart prompt | Health practitioners (physicians) | S: strong design and analysis  W: infrequent use of referrals limits meaningfulness of this outcome variable | - Increased IPV identification and referral rates |
| Harris, 2011 (UK) | Other (post-only, mixed method; CM) | Multimode without exchange: mailed educational resource with accompanying website | Health practitioners (dentists) | S: good sample size  W: methodological detail somewhat lacking | - most Ps reported improved knowledge and adoption of child protection policy  - over half Ps had identified child protection lead and a quarter had arranged further training as a result of the resource |
| Harris, 2002 | Experiment/RCT with pre-post (IPV & CM) | Multimode without exchange: 2-hour online educational program (links to materials, interactive case-based scenarios) | Health practitioners (physicians) | S: strong design, use of established measurement tool  W: some lack of detail regarding measures and intervention | - Improved confidence/self-efficacy, attitudes and self-reported knowledge regarding managing patients |
| Hawkins, 2001 (Australia) | Experiment/RCT (CM) | Unclear/unknown: 1 day training session | Teachers/educators | S: strong design and good methodological detail  W: insufficient detail regarding intervention | - Improved confidence (e.g., sign recognition), knowledge, and attitudes  - some Ps still reluctant to report based on suspicion of CM alone |
| Hazzard, 1984 (US) | Quasi-experimental pre-post (1 week) with 6 month follow-up (CM) | Multimode with exchange: 1 6-hour workshop (video, didactic presentation, small and large group discussion, role-play, modeling, question and answer with county protective services agency) | Teachers/educators | S: high response rate  W: some methodological detail lacking (e.g., recruitment) | - Improvements in knowledge regarding abuse and attitudes (empathy for abuse parents)  - Improvements in behaviour (e.g., increased discussion of abuse in class, decreased corporal punishment)  - No difference in number of abuse cases identified or reported |
| Heyman, 2009 (US) | Experiment/RCT (IPV & CM) | Complex/multifaceted: clinical chiefs - 1-hour online training and 2-day mock meeting, committee members - web training and competency quiz, compared phone vs. in-person consultant feedback | Other (family maltreatment determination committee members, e.g., social workers, police etc.) | S: design capable of distinguishing effectiveness of two components  W: some methodological detail lacking, unclear research setting | - agreement between field and master reviewer decisions good for most types of IPV and CM  - in-person training provided no significant benefit over phone |
| Hibbard, 1987 (US) | Pre-post (2 weeks) with 6-month follow-up (CM) | Multimode without exchange: 1 symposium (unknown duration; didactic presentation, discussions, role-play, demonstration) | Varied (e.g., caseworkers, physicians) | S: good description of intervention  W: some methodological details lacking, loss to follow-up | - Improvements in knowledge that were sustained at follow-up  - Increased use of dolls in interviewing  - No change in referral patterns |
| Hsieh, 2006 (US) | Experiment/RCT with pre-post (IPV and CM) | Multimode without exchange: 15-minute interactive multimedia tutorial | Health practitioners (dentists) | S: good methodological detail  W: post-test immediately following intervention, description of research setting lacking | - improved perceived knowledge and intentions to implement ADVR material (asking, validating, documenting, referring) compared to control  - less successful at changing attitudes and beliefs |
| Janssen, 2002 (Canada) | Other (post-only, multiple points of measurement; IPV) | Complex/multifaceted: 2 1-hour education sessions (second was more personal, e.g., recalling real life situations) assessment supervision, visual aids | Health practitioners (nurses) | S: strong theoretical underpinning  W: some methodological detail lacking (e.g., data collection and analysis) | - Screening rate increased at 6-month follow-up and was sustained at 18 months |
| Jones, 2004 (US) | Pre-post (immediate) with 3-year follow-up (CM) | Multimode without exchange: mail-out 5-videotape series with accompanying written instructional material | Health practitioners (physicians) | S: good description of research setting and problem  W: small sample, high loss to follow-up | - Improved knowledge in multiple areas (e.g., physical examination procedures)  - At follow-up, most Ps were still conducting sexual abuse examinations |
| Khan, 2005 (US) | Other (post-only; CM) | Unclear/unknown: 4-hour course | Health practitioners (physicians) | S: good sample size and sample size justification  W: insufficient detail regarding intervention and measurement tools | - self-reported increase in knowledge (e.g., signs of abuse), but not an increase in reporting of child abuse |
| Kleemeier, 1988 (US) | Experiment/RCT with pre-post (6-week follow-up; CM) | Multimode with exchange: 6-hour training workshop (didactic presentation, video, role-play, group discussion, question and answer with child protective services worker) | Teachers/educators | S: strong design, good intervention description  W: possible selection bias (experimental Ps more experience with abuse compared to controls) | - improved knowledge and attitudes at post-test compared to controls  - no difference between groups on prevention-related behaviours at follow-up |
| Knapp, 2006 (US) | Pre-post (immediate) with 6-month follow-up (IPV) | Multimode without exchange: 1 2-hour training session (video, case discussion, role-play) | Varied: health and social practitioners (e.g., physicians and social workers) | S: outcome measurement based on established tool  W: possible confounding factor of a screening program implemented after intervention but before follow-up | - Improved attitudes and self-efficacy regarding IPV screening  - Some self-reported IPV behaviour change at 6 months only |
| Lamb, 2000 (Israel) | Experiment/RCT with pre-post (CM) | Other: 4 conditions: 1) 1-week training (largely didactic), 2) 1-week training plus rapport-building training, 3) 2-day interview protocol training (role-play, discussion) with personal performance feedback and monthly refresher training, 4) perpetrator-focused protocol training, monthly refresher training | Social practitioners (forensic interviewers) | S: strong design, thorough descriptions of intervention conditions and coding of outcome measures  W: lacking detail regarding recruitment | - dramatic improvement in investigative child interview quality after intensive training (conditions 3 and 4)  - ongoing personalized feedback (condition 3) did not yield additional benefit |
| Larrivée, 2012 (Canada) | Systematic review (IPV/CM; quantitative and qualitative studies included) | Varied (13 ‘implementation studies’ reviewed) | Varied (e.g., health/social practitioners, community partners, etc.) | S: strong review of relevant literature, clear research questions  W: some methodological detail lacking | - Training to improve IPV/CM knowledge or attitudes effective in the short-term  - findings regarding effectiveness to change practice/behaviour are mixed  - Need for more diverse and rigorous assessment of KT strategies identified |
| Lia-Hoagberg, 1999 (US) | Other (IPV & CM; mixed method, 6-9 month post-test) | Passive/didactic: 1 training session (unspecified length) | Varied: public health nurses, public health agency directors | S: good theoretical underpinning, mixed method approach  W: low response rate, scant details on qualitative methodology and analysis | - Ps viewed the practice guidelines as important, but practical constraints make actual use challenging  - nurses with no use/intention to use guidelines were in minority |
| Lo Fo Wong, 2006 (Netherlands) | Experiment/RCT (IPV) | Multimode with exchange: compared 1.5 hour focus group (structured discussions) vs. focus group plus 1.5 day training (role-play, vignettes) vs. control | Health practitioners (physicians) | S: good justification of sample size, design capable of distinguishing effective intervention components  W: lacking detail regarding research setting | - Training improved IPV awareness (i.e., suspicion in the case of non-obvious signs) and identification  - Focus group alone doubled rate of active IPV questioning |
| Louwers, 2010 (Netherlands) | Systematic review (CM) | Varied (4 articles reviewed) | Health practitioners (e.g., physicians, nurses) | S: clear inclusion criteria, multiple search methods  W: no rationale for approach to quality appraisal, appraisal appears limited | - none of the interventions led to increased number of confirmed cases  - flowcharts/checklists identified as effective at improving suspicion rates, documentation and staff awareness |
| McCosker, 1999 (Australia) | Pre-post (mixed method; IPV) | Multimode with exchange: 8-hour distance education package (readings, activities, audio-taped interviews, mentor and teleconference support) | Health practitioners (e.g., nurses) | S: mixed method approach, description of intervention development  W: lacking some methodological detail (e.g., qualitative data collection and analysis) | - Improved knowledge and attitudes  - Some practice change reported such as better advocacy and increased community involvement |
| McGrath, 1987 (Canada) | Experiment/RCT with pre-post (immediate, 2-month follow-up; CM) | Multimode without exchange: 2-hour workshop based on manual (reading material, lectures, overheads, audio/visual resources) | Teachers/educators | S: use of established measurement tool, good description of research setting  W: high attrition, lacking methodological and analysis detail | - Improved knowledge (e.g., regarding board policy, warning signs of abuse), maintained at follow-up |
| Newton, 2010 (Canada) | Systematic review (CM) | Varied (6 studies reviewed) | Health practitioners (e.g., physicians, nurses) | S: comprehensive search strategy, good methodological detail  W: actual search terms not provided | - several moderate quality studies suggest education and reminder systems can improve knowledge and documentation, but this finding is not supported by a high quality RCT  - More (rigorously designed) research is needed to learn how to improve child abuse care in the ED setting |
| Nicolaidis, 2005 (US) | Pre-post (IPV) | Multimode with exchange: 2-hour session (video, advocate-led discussion, free resource materials) | Health practitioners (e.g., physicians, nurses) | S: good description of author-developed/ piloted measurement tool, rationale for intervention approach  W: possible selection bias, some analysis detail lacking | - Improved attitudes (e.g., responsibility, empathy), knowledge and self-reported behaviours (inquiry) |
| O’Campo, 2011 (Canada) | Systematic review (IPV; quantitative and qualitative studies included) | Varied (17 screening programs reviewed) | Varied: health and social practitioners (e.g., physicians, social workers) | S: thorough quality appraisal and methodological detail, clear inclusion criteria  W: actual search terms not provided | - ‘comprehensive’ programs tended to be effective at increasing IPV screening and identification rates  - ‘non-comprehensive’ programs did not incorporate multiple components and lacked institutional support |
| Olson, 1996 (US) | Pre-post (IPV) | Other: compared chart prompt to chart prompt plus 1-hour didactic training session | Varied (E.g., ED personnel, physicians, clerks, etc.) | S: design capable of distinguishing effectiveness of two components  W: some methodological detail lacking (e.g., recruitment) | - IPV identification rates increased after addition of chart prompt, education did not increase rates further |
| Paluzzi, 2000 (US) | Pre-post with follow-up (6 to 30 months depending on project component; mixed method; IPV) | Complex/multifaceted: Basic education – 2-day train-the-trainer course, ongoing technical support, site visits, Continuing education – 1-day training offered 4 times/year in various regions, 2 1-day training sessions, home study module with accompanying videos | Health practitioners (midwives) | S: thorough description of project development, consideration of replicability  W: high loss to follow-up, qualitative component of mixed method approach unclear | - Improved knowledge, attitudes and behaviours related to IPV screening |
| Paranal, 2012 (US) | Quasi-experimental pre-post (immediate) with follow-ups at 2 and 6 months (CM) | Multimode without exchange: online 3-hour training module (video, quizzes, etc.) | Varied employees/ volunteers from child-serving organizations (e.g., educators, religious leaders, counsellors, etc.) | S: rationale for intervention approach described, focus on process  W: methodological detail lacking, comparison group analyses not reported, main effectiveness findings based on organizational-level, retrospective reports | - improvements in self-reported behavioural outcomes (e.g., discussing child abuse) and understanding (e.g., of child sexual abuse and related policies)  -Ps felt emotionally supported by available resources during module |
| Protheroe, 2004 (UK) | Other (post-only, qualitative interviews; IPV and CM) | Multimode without exchange: 3-hour training session, 2- to 3-month reflective practice, 1-day training session | Health practitioners (midwives) | S: qualitative method and analysis well-described  W: poor response rate (possible selection bias), some intervention detail lacking | - Ps reported improved understanding of IPV, increased likelihood of identifying and supporting women  - considerable uncertainty regarding confidentiality and documentation |
| Rheingold, 2012 (US) | Experiment/RCT (CM) | Other: compared 2.5 hour in-person vs. web-based training (both included video, in-person with facilitated discussion) | Varied (e.g., child care professionals, teachers, day care providers, clergy, coaches) | S: thorough description of intervention, design capable of distinguishing different training formats  W: rationale for measurement tools used unclear | - both web and in-person Ps scored high on likelihood of using and sharing material with others  - web Ps experience greater discomfort during training, in-person Ps felt more supported |
| Rischke, 2011 (Canada) | Other (CM; pre-, mid- and post-intervention measurement) | Complex/multifaceted: 2-day training, written/verbal performance feedback, 2-day refresher training (training review and role-play), additional written-verbal feedback | Varied (child protection workers and police officers) | S: design capable of distinguishing effectiveness of components, thorough description of methods and analysis  W: small number of interviewees trained (but adequate sample of interviews coded), possible selection bias | - improvements in investigative child interview skills not seen until after refresher training |
| Salmon, 2006 (UK) | Pre-post (immediate) with 6-month follow-up (IPV) | Multimode with exchange: 1-day (7.5-hour) training (teaching and group work, role-play, case study) | Health practitioners (midwives) | S: good description of intervention, thorough outcome measures with some attempt to pilot  W: some aspects of analysis unclear | - Improved awareness, confidence (responding to IPV), and attitudes (e.g., stereotype reduction), changes declined at follow-up but remained above pre-test levels  - rates of enquiry lower than expected (most asked half the time) |
| Saunders, 2005 (US) | Quasi-experiment (mixed method; IPV) | Unclear/unknown: 1-day training session | Social practitioners (welfare caseworkers) | S: unique measurement of caseworker behaviour from perspective of female clients, good description of setting and problem  W: insufficient detail regarding intervention | - Trained workers more likely to discuss fear/harm with women, develop safety plan and be viewed by women as helpful |
| Schoening, 2004 (US) | Quasi-experiment with pre-post (IPV) | Multimode with exchange: compared 1-hour (video, powerpoint, presentation from local advocates) vs. 3-hour training (greater discussion/interaction, extra content) | Health practitioners (nurses) | S: use of established measurement tool, design capable of distinguishing effectiveness of different lengths of training, theoretical underpinning  W: poor response rate, possible selection bias (Ps chose which training to attend) | - Attitudes improved after 1-hour session if Ps had previous IPV education, and after 3-hour session if no previous education |
| Shefet, 2007 (Israel) | Pre-post (6 months; IPV and CM) | Multimode with exchange: 3 1-day workshops (elder, child, partner abuse; role-play, review and discussion of recorded scenarios) | Health practitioners (e.g., physicians) | S: novel, well-described intervention  W: high loss to follow-up, possible selection bias, some aspects of analysis unclear | - Improved knowledge, perceived skills, and decreased perceived barriers to responding to abuse  - Increased self-reports of routine screening and improvement on other actions (e.g., documentation) |
| Short, 2006 (US) | Experiment/RCT with pre-post (6 months, 12-month follow-up; IPV) | Multimode without exchange: 4-hour online continuing education program (interactive multimedia, case-based) | Health practitioners (physicians) | S: strong design, thorough description of intervention including development and rationale  W: loss to follow-up, self-report only | - Improved knowledge, attitudes (e.g., self-efficacy), self-reported behaviours (e.g., IPV management practices), maintained at follow-up |
| Smeekens, 2011 (Netherlands) | Experiment/RCT with pre-post (CM) | Multimode without exchange: 2-hour online learning program (simulated cases, videos, interactive elements) | Health practitioners (nurses) | S: theoretical underpinning, performance in case-based simulations as outcome measure  W: small sample size | - Improved self-efficacy and performance in simulated interviews  - Higher self-efficacy associated with better performance on child abuse detection simulations |
| Socolar, 1998 (US) | Experiment/RCT with pre-post (CM) | Other: personalized feedback on chart documentation quality, 4 standard articles to read, plus targeted articles | Health practitioners (physicians) | S: good description of intervention  W: engagement with intervention (e.g., reading articles) unknown | - Both groups improved documentation and knowledge pre to post; intervention had no effect |
| Sullivan, 1990 (US) | Experiment/RCT with pre-post (immediate, 3- and 6-month follow-ups, all groups received intervention; CM) | Multimode without exchange: 1-day training session (lecture, group discussion, experiential practice exercises) | Varied (e.g., nurses, social service personnel) | S: intervention well-described, rationale for study design provided  W: high loss to follow-up in some conditions, limited/unclear outcome measures | - Increased knowledge (e.g., how to facilitate disclosure)  - Increased number of reports made at follow-up by nonmedical staff, but not by medical staff |
| Thompson, 2000 (US) | Experiment/RCT with pre-post (9-10 months, follow-up at 21-23 months; IPV) | Complex/multifaceted: 2 half-day training sessions, extra training for designated leaders, newsletter, systems support and feedback, ‘environmental orchestration’ (e.g., posters), chart prompt | Health practitioners (e.g., physicians, nurses) | S: strong design, use of established measurement tool, good methodological detail  W: methodological and analytical approach to secondary, qualitative evaluation component unclear | - Increase in documented asking about IPV at 9 months  - decreased perceived barriers (e.g., fear of offense), increased self-efficacy and perceived asking (maintained at follow-up except for perceived asking) |
| Walker, 2009 (US) | Pre-post (CM) | Multimode without exchange: some 2-hour, some 1-day workshops (video with reference manual, presentation, development of community resource guide) | Varied (e.g., teachers, nurses, counselors) | S: good sample size and description of research setting and project development  W: lacking detail regarding measurement tool, analysis, and recruitment process | - Improved knowledge, self-reported skills and attitudes |
| Wathen, 2011 (Canada) | Other (IPV; longitudinal/ cross-sectional; mixed method) | Multimode with exchange: half-day workshops (research presentations, small/large group discussions), 1-day forum (research presentations, small/large group discussions) | Varied (e.g., researchers, health and social services leaders, policymakers) | S: strong, mixed method approach  W: lacking some recruitment details, self-report only | - types of knowledge use changed across time and were related to the types of decisions being made and the stage of decision-making  - most reported use was conceptual/ symbolic rather than instrumental |
| Warburton, 2006 (UK) | Pre-post (immediate; IPV) | Unclear/unknown: 2-hour seminar | Varied (e.g., clinical support workers, dentists, administrators) | S: comprehensive, established measurement tool used  W: intervention details (modes of delivery) lacking | - Improved attitudes, knowledge, self-efficacy and understanding of dental team’s role |
| Whitaker, 2012 (US) | Other (CM) | Complex/multifaceted: 5-day workshop (video, quizzes, role-play, modeling skills), coached field work | Social practitioners (e.g., child welfare workers) | S: evidence-based, well-described intervention, attempt to assess fidelity and connect mid-intervention scores with outcomes  W: recruitment of Ps (not agencies) unclear, post-only measurement, missing data | - intervention effective in increasing knowledge and resulted in good quality implementation of the SafeCare parenting program, but was not implemented often by Ps |
| Young, 2008 (US) | Quasi-experiment with pre-post (IPV and CM) | Multimode without exchange: 1.5-hour training (video, presentations) | Varied (e.g., teachers, administrative and cafeteria staff) | S: intervention well-described, good sample size  W: limited outcome measure | - Each occupational group showed improved knowledge (e.g., signs, identification of community resources)  - Some groups (e.g., nurses) who may have had previous training had smaller knowledge gains |
| Zachary, 2002 (US) | Other (pre-post with focus groups; mixed method; IPV) | Complex/multifaceted: 2 didactic training sessions, chart prompt, onsite domestic violence coordinator | Health practitioners (e.g., physicians, nurses) | S: mixed method approach, established measurement tool used  W: small sample, some methodological details lacking | - Improved self-efficacy and self-reported behaviours (e.g., referring)  - Little improvement in overall knowledge and attitudes |

Note: The articles may report on other initiatives and findings not summarized in this table. Only aspects of the research most relevant to our review are summarized. Study design is quantitative and experiments/RCTs compared intervention group to at least one control (i.e., no intervention) group, unless otherwise noted. Methodological weaknesses inherent to particular studies designs (e.g., no control group, lack of long-term follow-up) are not reiterated here.
