# Supplementary material for: Strategies to promote uptake and use of intimate partner violence and child maltreatment knowledge: an integrative review
Source: BMC Public Health. 2014 Aug 21;14:862. doi: 10.1186/1471-2458-14-862 (PMC4152574; doi:10.1186/1471-2458-14-862)
Supplement: Supplementary file 4 — Additional file 4: Quality Appraisal for 52 Excluded Articles.(DOCX 27 KB) [file 12889_2014_6991_MOESM4_ESM.docx]

Additional File 4: Quality Appraisal for 52 Excluded Articles

| **Excluded Articles** |  |
| --- | --- |
| **Article/Reference** | **Key Strengths (S) & Weaknesses (W)** |
| 1. Steen M, Bharj K: **Midwives’ reflections: exploring attitudes, feelings and experiences when caring for women who are being abused.** *Midirs* 2003, **13**(1):115-118. | S: Novel intervention, large sample  W: Insufficient details regarding methods (e.g., recruitment, coding) and analysis, some details regarding intervention lacking |
| 1. Kim J, Motsei M: **“Women enjoy punishment”: Attitudes and experiences of gender-based violence among PHC nurses in rural South Africa.** *Soc Sci Med* 2002, **54**(8):1243-1254. | S: Project rationale clear, mixed method approach  W: Small sample, insufficient detail regarding intervention, methods, recruitment, and analysis |
| 1. Needleman HL, MacGregor SS, Lynch LM: **Effectiveness of a statewide child abuse and neglect educational program for dental professionals.** *Pediatr Dent* 1995, **17**(1):41-45. | S: Large sample  W: Insufficient methodological detail (e.g., measures, intervention) |
| 1. Volpe R: **The development and evaluation of a training program for school-based professionals dealing with child abuse.** *Child Abuse Negl* 1981, **5**(2):103-110. | S: Thorough intervention description  W: Insufficient detail regarding methods (e.g., measures) and analysis, small sample size |
| 1. Brackley MH: **Safe family project: a training model to improve care to victims of domestic violence.** *J Nurses Staff Dev* 2008, **24**(1):E16-27. | S: Good description of intervention content  W: Insufficient detail regarding methods (e.g., measures, sample) and analysis |
| 1. Ramsden C, Bonner M: **An early identification and intervention model for domestic violence.** *Aust Emerg Nurs J* 2002, **5**(1):15-20. | S: Measured behavioural outcomes (referral and screening rates)  W: Insufficient detail regarding methods (e.g., data collection) and analysis, conclusions not well supported by purely descriptive analyses |
| 1. Anderson SE, Youngson SC: **Introduction of a child sexual abuse policy in a health district.** *Child Soc* 1990, **4**(4):401-419. | S: Novel, behavioural outcome variable (self-reported teaching sessions held)  W: Insufficient detail regarding methods (e.g., recruitment) and analysis |
| 1. Weyts A, Morpeth L, Bullock R: **Department of Health research overviews - past, present and future: An evaluation of the dissemination of the Blue Book, Child Protection: Messages from research.** *Child Fam Soc Work* 2000, **5**(3): 215-23. | S: Detailed historical context provided  W: Low response rate, insufficient detail regarding data collection, measures, and analysis |
| 1. Swenson-Britt E, Thornton JE, Hoppe SK, Brackley MH: **A continuous improvement process for health providers of victims of domestic violence.** *Joint Comm J Qual Im* 2001, **27**(10):540-554. | S: Established measurement tool used  W: Insufficient detail and lack of clarity regarding methods, intervention and analysis |
| 1. Allsopp A, Prosen S: **Teacher reactions to a child sexual abuse training program.** *Elem School Guid Couns* 1988, **22**(4):299-305. | S: Good description of intervention content  W: Insufficient detail regarding methods and analysis, inadequate measurement tool, descriptive analysis of pre-post data only |
| 1. Bokunewicz B, Copel LC: **Attitudes of emergency nurses before and after a 60-minute educational presentation on partner abuse.** *J Emerg Nurs* 1992, **18**(1):24-27. | S: Good description of intervention content  W: Low response rate, insufficient detail regarding measures and analysis, inadequate outcome measures |
| 1. Boursnell M, Prosser S: **Putting children in the picture: Improving responses to domestic violence in the emergency department.** *Developing Pract* 2009, **23**:56-63. | S: Mixed method approach  W: Insufficient detail regarding intervention and measures, high loss to follow-up, pre-post data analyzed descriptively |
| 1. Campbell H, Macdonald S: **Child protection in schools: An evaluation of a training course for Fife schools’ co-ordinators of child protection.** *Public Health* 1996, **110**(1):37-40. | S: Mixed method approach  W: Small sample size, insufficient detail regarding intervention, methods, and analysis |
| 1. Carter YH, Bannon MJ, Limbert C, Docherty A, Barlow J: **Improving child protection: A systematic review of training and procedural interventions.** *Arch Dis Child* 2006, **91**(9):740-743. | S: Many databases searched  W: Search strategy not comprehensive (e.g., reference lists not searched), database search terms not provided, quality appraisal not used, insufficient detail regarding research questions and inclusion/ exclusion criteria |
| 1. Chez RA, Horan DL: **Response of obstetrics and gynecology program directors to a domestic violence lecture module.** *Am J Obstet Gynecol* 1999, **180**:496-498. | S: Collected data regarding useful content for future interventions  W: Insufficient detail regarding intervention, methods and analysis, inadequate outcome measure, poor response rate |
| 1. Cowley S, Rush J, Lenton RL, Lukasik-Foss L: **Care of the abused woman. A hospital’s educational program assesses values and beliefs.** *Health Prog* 1996, **77**(2):26-29. | S: Good sample size  W: Insufficient detail regarding intervention, methods, and analysis |
| 1. Davis JW, Kaups KL, Campbell SD, Parks SN: **Domestic violence and the trauma surgeon: Results of a study on knowledge and education.** *J Am Coll Surg* 2000, **191**(4):347-353. | S: Quasi-experimental design with pre-post  W: Insufficient detail regarding intervention, methods and analysis |
| 1. Delewski CH, Pecora PJ, Smith G, Smith V Jr.: **Evaluating child protective services training: the participant action plan approach.** *Child Welfare* 1986, **65**(6):579-591. | S: Rationale behind intervention and evaluation approach provided  W: Low response rate, insufficient detail regarding methods and analysis |
| 1. Dixon CK: **Clergy as carers: A response to the pastoral concern of violence in the family.** *J Psychol Christianity* 1997, **16**(2):126-131. | S: Thorough intervention description  W: Insufficient detail regarding methods (e.g., sample, recruitment, measures) and analysis |
| 1. Haney K, Kachur E, Zabar S: **A brief but multi-faceted approach improves clinicians’ domestic violence confidence, competence and clinical performance.** *Med Educ* 2003, **37**(5):488-489. | S: Good description of intervention format  W: Small sample size, insufficient detail regarding methods and analysis |
| 1. Harmer-Beem M: **The perceived likelihood of dental hygienists to report abuse before and after a training program.** *J Dent Hyg* 2005, **79**(1):7. | S: Details regarding outcome measure and analyses provided in full  W: Small sample size, insufficient detail regarding intervention, inadequate outcome measure |
| 1. Hawkins R, McCallum C: **Effects of mandatory notification training on the tendency to report hypothetical cases of child abuse and neglect.** *Child Abuse Rev* 2001, **10**(5):301-322. | S: Adapted version of established measure used, strong design  W: Low response rate, insufficient detail regarding intervention |
| 1. Helton A, McFarlane J, Anderson E: **Prevention of battering during pregnancy: Focus on behavioral change.** *Public Health Nurs* 1987, **4**(3):166-174. | S: Theoretical underpinning of intervention provided  W: Insufficient detail regarding measures and analysis |
| 1. Henry BM, Ueda R, Shinjo M, Yoshikawa C: Health **education for nurses in Japan to combat child abuse.** *Nurs Health Sci* 2003, **5**(3):199-206. | S: Thorough intervention description  W: Inadequate outcome measures, insufficient detail regarding recruitment (possible selection bias) |
| 1. McFarlane J, Anderson ET, Helton A: **Response to battering during pregnancy: An educational program.** *Response Victimization Women Children* 1987, **10**(2):25-26. | S: Pre-post design with sub-analyses by profession  W: Limited outcome measures, insufficient detail regarding intervention, methods (sample size, recruitment, measures), and analysis |
| 1. Milne D, Harrison L: **Dealing with child abuse: Development and evaluation of a short workshop for nurses.** *Nurse Educ Today* 1993, **13**(5):389-392. | S: Multiple points of measurement  W: Insufficient detail regarding analysis, some methodological details lacking (e.g., recruitment) |
| 1. Nelms TP: **An educational program to examine emergency nurses’ attitudes and enhance caring intervention with battered women.** *J Emerg Nurs* 1999, **25**(4):290-293. | S: Good description of intervention content  W: Insufficient detail regarding methods (e.g., recruitment, measures) and qualitative analysis |
| 1. Polnay J, Blair M: **A model programme for busy learners.** *Child Abuse Rev* 1999, **8**(4):284-288. | S: Thorough intervention description  W: Limited outcome measures, insufficient detail regarding methods and analysis |
| 1. Schroeder M, Weber JR: **Promoting domestic violence education for nurses.** *Nurs Forum* 1998, **33**(4):13-21. | S: Thorough intervention description  W: Small sample size, limited outcome measures, insufficient detail regarding analysis, conclusions not well-supported by findings |
| 1. Turner S: **Keep children safe.** *Nurs Stand* 2009, **23**(25):62-63. | S: Rationale behind intervention approach provided  W: Insufficient details regarding methods (sample size, measures, etc.) and analysis |
| 1. Wallace A: **Domestic violence: an education programme for hospital staff.** *J Fam Health Care* 2002, **12**(3):65-67. | S: Development of intervention described  W: Small sample size, insufficient details regarding methods and analysis, limited outcome measures |
| 1. Weiss SJ, Ernst AA, Blanton D, Sewell D, Nick TG: **EMT domestic violence knowledge and the results of an educational intervention.** *Am J Emerg Med* 2000, **18**(2):168-171. | S: Adequate description of intervention content  W: High loss to follow-up, small sample size, limited outcome measure |
| 1. Welbury RR, Hobson RS, Stephenson JJ, Jepson NJ: **Evaluation of a computer-assisted learning programme on the oro-facial signs of child physical abuse (non-accidental injury) by general dental practitioners.** *Brit Dent J* 2001, **190**(12):668-670. | S: Good description of intervention format  W: Some details of methods (e.g., measure properties) and analysis lacking, some aspects of analysis unclear, limited outcome measures |
| 1. Young A, McFarlane J: **Preventing abuse during pregnancy: A national educational model for health providers.** *J Nurs Educ* 1991, **30**(5):202-206. | S: Theoretical underpinning of intervention approach described  W: Small sample size, insufficient detail regarding methods (e.g., measures) and analysis |
| 1. Weir A, Lynch E, Hodes DT, Goodhart CL**: The role of the GP in child protection and family support: A collaborative training model.** *Child Abuse Rev* 1997, **6**:65-9. | S: Adequate description of intervention content  W: Insufficient details regarding methods and analysis, limited outcome measures, low response rate |
| 1. Barker J: **Extending the scope of child protection training.** *Child Abuse Rev* 1998, **7**:287-93. | S: Good description of research setting  W: Insufficient details regarding intervention, methods (e.g., sample size, measures), and analysis, conclusions not well-supported by findings |
| 1. Bhrolchain C, Shribman S, Hales V: **Training the practice health care team.** *Child Abuse Rev* 1995, **4**:83. | S: Adequate description of intervention format  W: Small sample size, evaluation component very limited, insufficient details regarding methods and analysis |
| 1. Buckley H: **Working together to protect children: evaluation of an interagency training programme.** *Admin* 2000, **48**:24-42. | S: Good description of research setting  W: Evaluation component very limited, insufficient details regarding methods and analysis |
| 1. Burton D, Stanley D, Ireson C: **Child advocacy outreach: Using telehealth to expand child sexual abuse services in rural Kentucky.** *J Telemed Telecare* 2002, **8**(suppl 2):10-12. | S: Measured process variables  W: Small sample size, insufficient detail regarding methods and analysis |
| 1. Myers JL: **Workshop effectiveness: Nurses as witnesses in court cases involving physical child abuse.** *J Nurs Law* 1996, **3**:35-44. | S: Adequate detail regarding intervention format  W: Limited outcome measures, insufficient detail regarding methods (e.g., measures) and analysis |
| 1. Polnay J, Curnock D: **What’s in a name? Named doctor in child protection - interpretation and implementation of the role.** *Child Abuse Rev* 2003, **12**:335-46. | S: Good description of research setting  W: Insufficient detail regarding intervention, methods (e.g., sample size), and analysis |
| 1. Brewer H, Mitchell C, Tomlinson R: **A child protection week: An effective method of hospital-wide training.** *Child Abuse Rev* 2012, **21**:362-369. | S: Novel intervention  W: Low response rate, limited outcome measures, insufficient detail regarding analysis |
| 1. Davis RE, Harsh KE: **Confronting barriers to universal screening for domestic violence.** *J Prof Nurs* 2001, **17**(6): 313-320. | S: Attention to various types of barriers  W: Insufficient detail regarding intervention, methods (e.g., data collection) and analysis (results are largely informal) |
| 1. Berman S, Barlow KA, Koziol-McLain J: **Family violence prevention education programme for midwives: An Auckland evaluation.** New Zeal Coll Midwives J 2005, **32**:21-26. | S: Measured intervention process, explicit learning objectives  W: Low response rate, small sample size, insufficient details regarding qualitative analysis |
| 1. Minsky-Kelly D, Hamberger LK, Pape DA, Wolff M: **We’ve had training, now what? Qualitative analysis of barriers to domestic violence screening and referral in a health care setting.** *J Interpers Violence* 2005, **20**(10):1288-1309. | S: Good qualitative methodology  W: Focus groups not audio-recorded, results regarding barriers not clearly linked to intervention, sub-analyses by department presented but n unknown |
| 1. Pagel JR, Pagel PR: **Participants’ perceptions of a mandated training course in the identification and reporting of child abuse.** *Pediatr Nurs* 1993, **19**(6): 554-558. | S: Good sample size  W: Limited outcome measures, insufficient details regarding methods (e.g., recruitment) and qualitative analysis |
| 1. Perrin KM, Boyett TR, McDermott RJ: **Continuing education about physically abusive relationships:** **Does education change the perceptions of health care practitioners?** *J Contin Educ Nurs* 2000, **31**(6): 269-274. | S: Full statistics reported  W: Low response rate, limited outcome measures, insufficient detail regarding intervention and methods |
| 1. Power C, Bahnisch L, McCarthy D: **Social work in the emergency department—Implementation of a domestic and family violence screening program.** *Aust Soc Work* 2011, **64**(4):537-554. | S: Good description of research setting and project development  W: Low response rate, small sample size, insufficient detail regarding intervention, lacking some details regarding methods and analysis |
| 1. Price S, Baird K, Salmon D: **Does routine antenatal enquiry lead to an increased rate of disclosure of domestic abuse? Findings from the Bristol Pregnancy and Domestic Violence Programme.** *Evid Based Midwifery* 2007, **5**(3):100-106. | S: Strong design, mixed method approach  W: Insufficient detail regarding intervention (content, duration and format unknown) |
| 1. Ramsden C, Bonner M: **A realistic view of domestic violence screening in an emergency department.** *Accid Emerg Nurs* 2002, 10(1):31-39. | S: Good description of research setting  W: Insufficient detail regarding methods (e.g., sample size) and (especially qualitative) analysis |
| 1. Roark SV: **Intimate partner violence: Screening and intervention in the health care setting.** *J Contin Educ Nurs* 2010, **41**(11):490-495. | S: Good intervention description  W: Insufficient detail regarding methods (e.g., measures, sample size) and analysis |
| 1. Shattuck SR: **A domestic violence screening program in a public health department.** *J Community Health Nurs* 2002, **19**(3):121-132. | S: Theoretical underpinning of intervention provided  W: Small sample size, some detail regarding intervention lacking, limited outcome measures, insufficient detail regarding analysis |
| Note: Many of the ‘poor’ quality articles were likely not intended to be ‘research articles’ per se or did not have evaluation of an intervention as their primary purpose, and therefore did not contain adequate detail to receive a higher quality rating | |
