# Supplementary figures and images for: Strategies to promote uptake and use of intimate partner violence and child maltreatment knowledge: an integrative review
Source: BMC Public Health. 2014 Aug 21;14:862. doi: 10.1186/1471-2458-14-862 (PMC4152574; doi:10.1186/1471-2458-14-862)

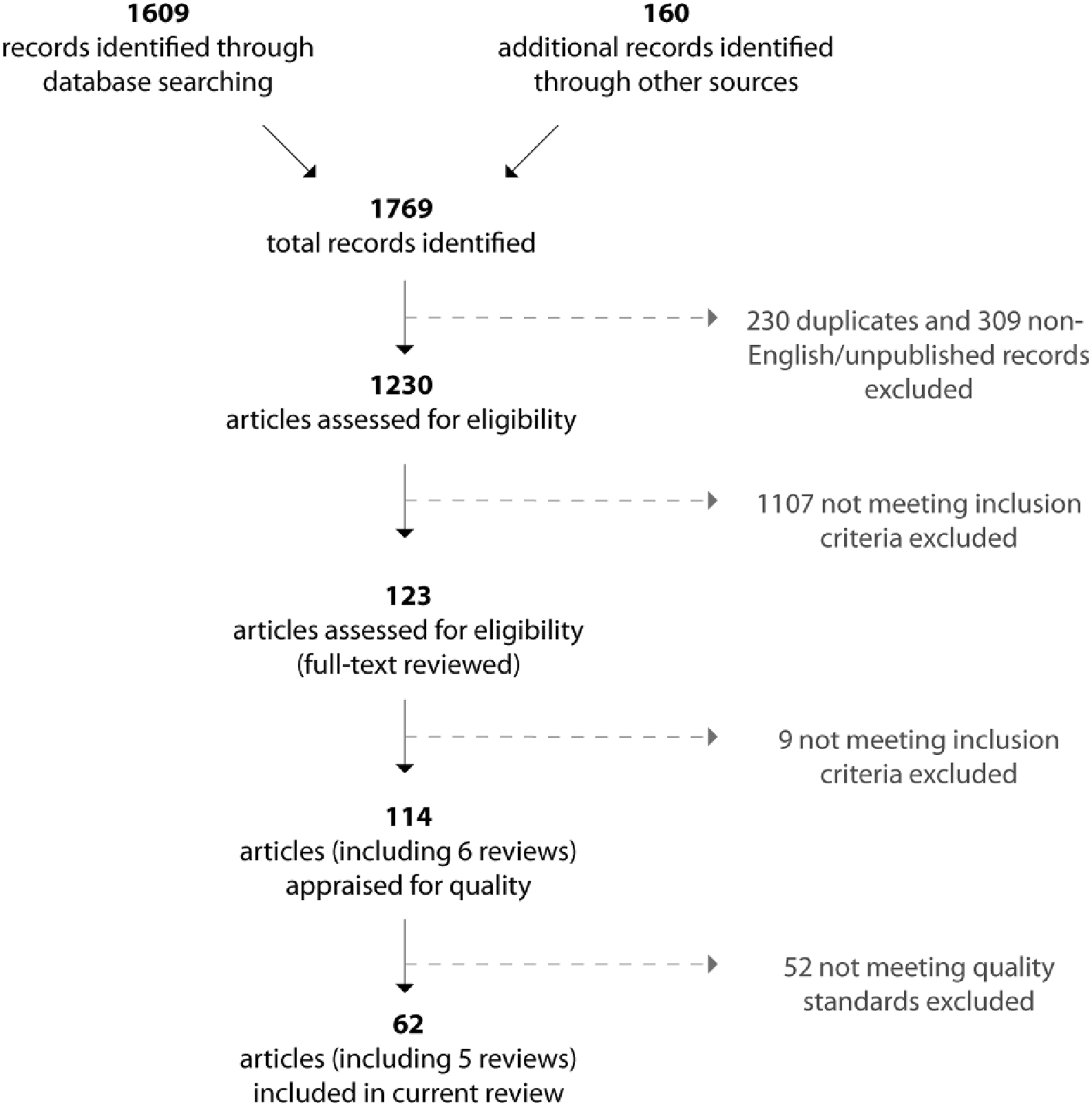

Supplement: Supplementary file 5 — Authors’ original file for figure 1 [file 12889_2014_6991_MOESM5_ESM.tif]
