# Supplementary material for: Strategies to promote uptake and use of intimate partner violence and child maltreatment knowledge: an integrative review
Source: BMC Public Health. 2014 Aug 21;14:862. doi: 10.1186/1471-2458-14-862 (PMC4152574; doi:10.1186/1471-2458-14-862)
Supplement: Supplementary file 6 — Authors’ original file for figure 2 [file 12889_2014_6991_MOESM6_ESM.pdf]

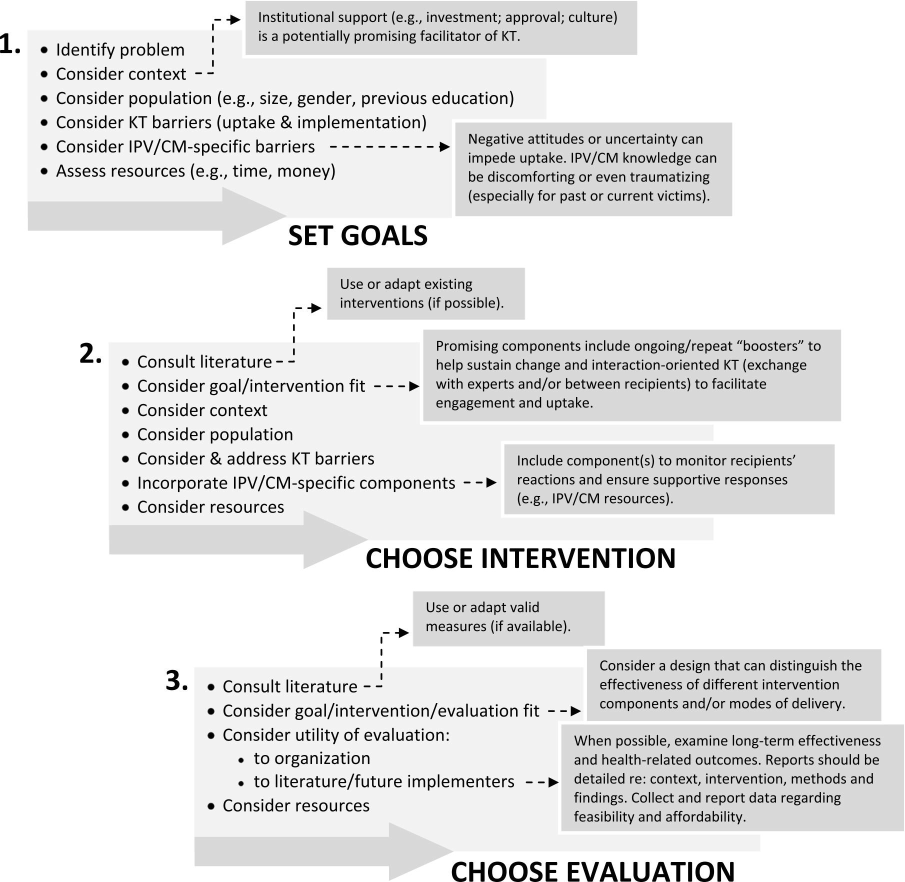
1.
Identify problem
Consider context
Institutional support (e.g., investment; approval; culture) is a potentially promising facilitator of KT.
Consider population (e.g., size, gender, previous education)
Consider KT barriers (uptake & implementation)
Consider IPV/CM-specific barriers
Negative attitudes or uncertainty can impede uptake. IPV/CM knowledge can be discomforting or even traumatizing (especially for past or current victims).
Assess resources (e.g., time, money)
SET GOALS
2.
Consult literature
Use or adapt existing interventions (if possible).
Consider goal/intervention fit
Promising components include ongoing/repeat “boosters” to help sustain change and interaction-oriented KT (exchange with experts and/or between recipients) to facilitate engagement and uptake.
Consider context
Consider population
Consider & address KT barriers
Incorporate IPV/CM-specific components
Include component(s) to monitor recipients’ reactions and ensure supportive responses (e.g., IPV/CM resources).
Consider resources
CHOOSE INTERVENTION
3.
Consult literature
Use or adapt valid measures (if available).
Consider goal/intervention/evaluation fit
Consider a design that can distinguish the effectiveness of different intervention components and/or modes of delivery.
Consider utility of evaluation:
to organization
to literature/future implementers
When possible, examine long-term effectiveness and health-related outcomes. Reports should be detailed re: context, intervention, methods and findings. Collect and report data regarding feasibility and affordability.
Consider resources
CHOOSE EVALUATION
